# Candidate chemoreceptor subfamilies differentially expressed in the chemosensory organs of the mollusc *Aplysia*

**DOI:** 10.1186/1741-7007-7-28

**Published:** 2009-06-04

**Authors:** Scott F Cummins, Dirk Erpenbeck, Zhihua Zou, Charles Claudianos, Leonid L Moroz, Gregg T Nagle, Bernard M Degnan

**Affiliations:** 1 School of Biological Sciences, The University of Queensland, Brisbane, Queensland 4072, Australia; 2 Department of Earth and Environmental Sciences, Ludwig-Maximilians-University, Munich, Germany; 3 Department of Neuroscience and Cell Biology, University of Texas Medical Branch, Galveston, TX 77555, USA; 4 Queensland Brain Institute, The University of Queensland, St. Lucia, Queensland, 4072, Australia; 5 Whitney Laboratory for Marine Science and Department of Neuroscience, University of Florida, St Augustine, Florida 32080, USA

## Abstract

**Background:**

Marine molluscs, as is the case with most aquatic animals, rely heavily on olfactory cues for survival. In the mollusc *Aplysia californica*, mate-attraction is mediated by a blend of water-borne protein pheromones that are detected by sensory structures called rhinophores. The expression of G protein and phospholipase C signaling molecules in this organ is consistent with chemosensory detection being via a G-protein-coupled signaling mechanism.

**Results:**

Here we show that novel multi-transmembrane proteins with similarity to rhodopsin G-protein coupled receptors are expressed in sensory epithelia microdissected from the *Aplysia *rhinophore. Analysis of the *A. californica *genome reveals that these are part of larger multigene families that possess features found in metazoan chemosensory receptor families (that is, these families chiefly consist of single exon genes that are clustered in the genome). Phylogenetic analyses show that the novel *Aplysia *G-protein coupled receptor-like proteins represent three distinct monophyletic subfamilies. Representatives of each subfamily are restricted to or differentially expressed in the rhinophore and oral tentacles, suggesting that they encode functional chemoreceptors and that these olfactory organs sense different chemicals. Those expressed in rhinophores may sense water-borne pheromones. Secondary signaling component proteins Gα_q_, Gα_i_, and Gα_o _are also expressed in the rhinophore sensory epithelium.

**Conclusion:**

The novel rhodopsin G-protein coupled receptor-like gene subfamilies identified here do not have closely related identifiable orthologs in other metazoans, suggesting that they arose by a lineage-specific expansion as has been observed in chemosensory receptor families in other bilaterians. These candidate chemosensory receptors are expressed and often restricted to rhinophores and oral tentacles, lending support to the notion that water-borne chemical detection in *Aplysia *involves species- or lineage-specific families of chemosensory receptors.

## Background

All animals must recognize and respond to chemosensory information in their environment. Although the marine mollusc *Aplysia *has been a valuable model to investigate the molecular basis of behavior [1,2] and reproduction [3,4], our knowledge of how they recognize and respond to environmental signals is limited. In particular, it is unknown how they distinguish and bind water-soluble molecules and transfer exogenous information intracellularly. In contrast, the molecular components and mechanisms of chemical detection in a range of vertebrates and other invertebrates have been well studied.

Vertebrate chemoreception is made possible by six distinct classes of multi-transmembrane receptors: (i) olfactory receptors (ORs) [5], (ii) trace amine-associated receptors [6], vomeronasal receptors (iii) type 1 and (iv) type 2 [7,8] and taste receptors (v) type 1 and (vi) type 2 [9,10]. Besides binding chemical molecules, all share the common traits of seven transmembrane (7-TM) domains, G-protein signaling and precise sensory cell expression. In mammals, non-volatile pheromone perception is thought to act primarily through the vomeronasal organ sensory epithelium [11] and be mediated intracellular via the interaction of chemical molecules with vomeronasal receptors located on the dendrites of vomeronasal sensory neurons [12]. However, in teleost fishes who do not have a vomeronasal organ, the vomeronasal receptors are found in the main olfactory epithelium [13]. It appears that genes involved in an animal's response to its environment are subject to extensive gene duplication, gene loss and lineage-specific expansion over time, leading to large gene families such as those observed in the OR and vomeronasal receptor repertoire. In fact, OR genes represent the largest mammalian gene family [14].

Chemoreception through 7-TM domain receptors appears to have evolved multiple times independently, as vertebrate chemoreceptors are not closely related to those known in insects and nematodes. Recognition of external chemicals in *Drosophila *is accomplished by families of 130 genes encoding 7-TM domain receptors [15,16], including OR (60) and gustatory receptors (70). Gustatory receptors are greatly reduced in the honeybee [17]. Insect chemoreceptors do not belong to the G-protein coupled receptor (GPCR) family due to a unique inverse membrane topology [18]. Rather, they use an alternative, non-G protein-based signaling pathway where receptors not only detect chemicals but can also act as ion channels [19]. In support of this, heterologous cells expressing silkmoth, fruitfly or mosquito heteromeric OR complexes showed G-protein independent extracellular calcium influx and cation-non-selective ion conductance upon stimulation with odorant [19]. Nevertheless, chemical detection is still mediated by a large and divergent family of 7-TM domain receptors.

A central issue that has not been adequately addressed is how water-borne chemicals are detected at the molecular level by the huge diversity of invertebrates that inhabit marine environments. In marine invertebrates, chemosensory abilities are essential for almost all aspects of their life, from feeding to predator avoidance and reproduction. A recent bioinformatic survey of the sea urchin genome resulted in the identification of a remarkable diversity of chemoreceptors, expressed specifically and differentially in adult sensory structures [20]. Meanwhile, there have been important findings forthcoming from research into the molluscan group. Olfactory studies of squid have shown that both phospholipase C (PLC) and cAMP-mediated pathways may be involved in olfactory sensory neurons activation [21]. In support of this, immunolocalization experiments revealed the presence of G proteins involved in both cAMP (Gα_o_) and PLC (Gα_q_) pathways which are clearly co-expressed in certain cell types.

*Aplysia *possesses many advantages necessary for chemical communication research, such as an extensive knowledge of its anatomy, a detailed understanding of the molecular and cellular basis of behavior, and now considerable genomic and expressed sequence tag (EST) resources. Moreover, we have found that in *Aplysia*, conspecific and congener attraction is mediated by a remarkable cocktail of water-borne protein pheromones [4,22]. In *Aplysia*, freshly laid egg cordons are considered to be a source of both water-borne and contact pheromones that attract conspecifics and closely related species to the area and induce them to mate and lay eggs. Egg laying results in the release of at least four proteinaceous attraction pheromones, including the 58-residue attractin [4,23,24]. T-maze bioassays have demonstrated that binary blends of attractin with either enticin, temptin or seductin are sufficient to attract potential mates [23].

At the anatomical level, *Aplysia *chemosensory detection is achieved by the rhinophore [25], specialized anterior sensory organs on the dorsal surface of the head. Rhinophore are retractile and primarily used for distance chemoreception and rheoreception (response to water current), whereas the oral tentacles, which are found more ventrally, are possibly involved in contact chemoreception and mechanoreception [26]. The neuroanatomical organization of rhinophores includes a rhinophore groove where most of the sensory cells appear to be concentrated. Its sensory epithelium contains sensory neurons that project axons back to rhinophore ganglia and dendrites that end in either a surface-exposed cilium or a small protuberance [26-28]. Consistent with a potential role in chemical transduction, gene transcripts encoding G protein, PLC or inositol 1,4,5-trisphosphate receptor were found to be expressed in *Aplysia *rhinophore sensory epithelium [29]. The involvement of nitric oxide as a potential chemosensory processing component has also been implicated in molluscan chemoreception based on cytological NADH-diaphorase histochemistry of the *Aplysia *rhinophore [30]. Of significance, was the finding that nitric oxide synthase is present in epithelial sensory-like cells that had multiple apical ciliated processes exposed to the environment. This is consistent with findings demonstrating that inhibition of nitric oxide synthase disrupts slime trail following, suggesting a role for nitric oxide in neural processing of stimuli in snails [31].

The presence of G protein mRNAs in *Aplysia *sensory epithelium suggested that multi-transmembrane GPCR-like proteins could play an important role in chemosensory detection. With the availability of a 2× genome coverage for *Aplysia californica*, we expected that it would provide an excellent and first opportunity to investigate the molecular basis of chemical detection in a mollusc. Here, we performed iterative Basic Local Alignment Search Tool (BLAST) searches to identify genes similar to rhodopsin GPCR genes encoding 7-TM domains from the *A. californica *genome. We identified genes representing three unique monophyletic families that show rhinophore, oral tentacle and ovotestis expression. Based on their expression, these may encode chemosensory proteins, including pheromone and gustatory receptors. Antisera directed against a conserved region of a candidate chemosensory receptor, as well as *Aplysia *Gα_q_, Gα_i_, and Gα_o_, confirmed their expression in sensory tissues, with localization to the outer sensory epithelium.

## Results

### Identification of genes encoding rhodopsin G-protein coupled receptor-like proteins in the Aplysia californica genome

We performed iterative tBLASTn for closely related novel genes and discovered a large number of genes encoding rhodopsin GPCR-like proteins. Using this approach, we successfully identified a total of 90 genes encoding proteins belonging to the GPCR superfamily. Of these, 72 were predicted to contain 7-TM domains. It was not possible to annotate the full-length sequence of all genes, especially in the 5'-regions, and 18 genes encoding six transmembrane domains were considered partial-length. Note that these numbers are the minimal estimates, because the genome sequencing of *A. californica *had not been fully completed. We expect that further rhodopsin GPCR-like genes will be found beyond the ones we have identified. Also, several of the multi-transmembrane gene models appeared to be pseudogenes with various defects, including the insertion of stop codons and frame-shifting indels leading to premature termination of the coding region. These were not included in the final data set.

### Phylogenetic construction and analysis of identified rhodopsin G-protein coupled receptor-like genes

**Figure 1:**
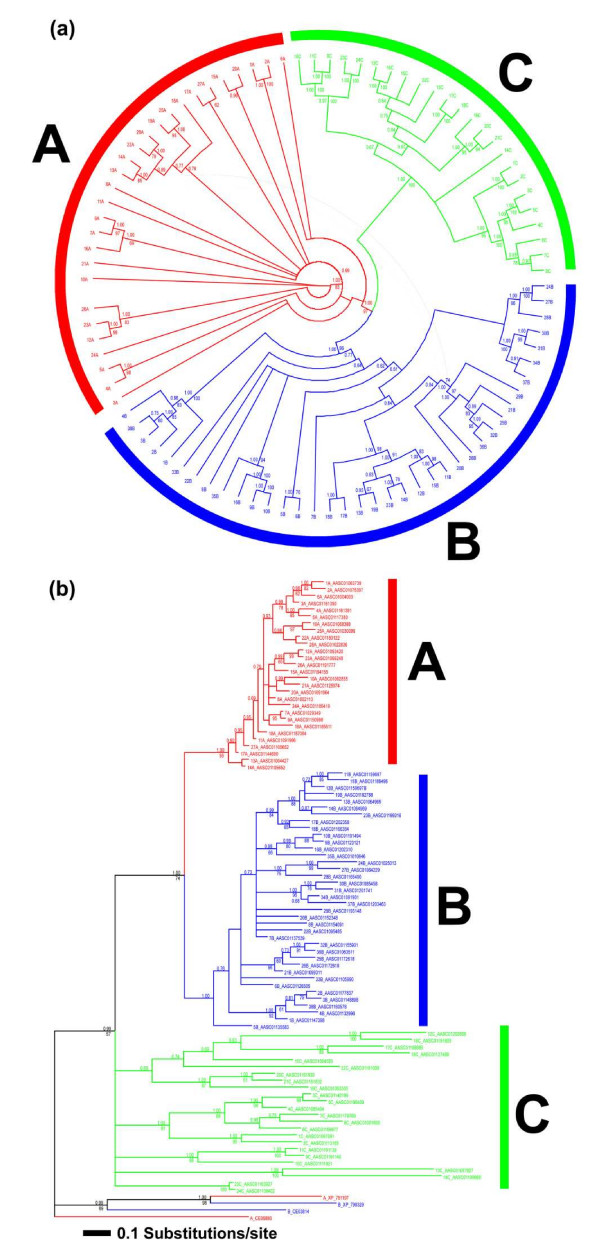
**Phylogenetic reconstruction of identified *Aplysia *rhodopsin G-protein coupled receptor-like gene sequences**. Subfamily type a (red) type b (blue) and type c (green). The trees are based on the bayesian inference reconstruction combined with the RAxML results. The numbers at the branches correspond to posterior probabilities (>75) and congruent bootstrap probabilities (>60, with exceptions), respectively. **(a) **Unrooted tree without non-*Aplysia *sequences. **(b) **Phylogram with non-*Aplysia *sequences included.

We have performed phylogenetic analyses of the 90 selected rhodopsin GPCR-like genes (ingroup), together with four non-*Aplysia *GPCR genes (outgroup). The 94 sequences (including outgroup taxa) comprised 264 to 444 characters. For the two phylogenetic analyses (with and without outgroup, respectively) we had to restrict the character sets to 96 and 166 alignable positions, respectively, in order to maintain the conservative approach. Both phylogenetic reconstructions, with and without outgroup, display a congruent picture regarding the phylogenetic relationship of the *Aplysia *GPCRs (Figures 1a and 1b). Branch lengths of groups A (subfamily a) and B (subfamily b), especially in the former, are considerably shorter than of group C (subfamily c). GPCR-like gene group features are summarized in Additional file 1. The phylogenetic analyses support a monophyly of the three different subfamily groups, although sufficient phylogenetic signal for subfamily c monophyly is achievable only when non-*Aplysia *taxa are not present.

Besides structural predictions that place these genes within the 7-TM superfamily, which includes rhodopsin GPCRs, these genes have little amino acid identity (<10%) to any known genes, and do not appear in the published *Aplysia *EST neuronal transcriptome [32]. They show only distant similarity with known molluscan multi-transmembrane receptors, including well-characterized *Aplysia *neurotransmitter GPCRs. GenBank tBLASTn searches also reveal most amino acid identity with regions of orphan GPCRs of the sea urchin, *Strongylocentrotus purpuratus *(E value 1e-11), various ghrelin receptors (for example, *Rattus norvegicus*, E value 2e-07) and a candidate GPCR (*Caenorhabditis elegans*, E value 4e-04) for subfamilies a, b and c, respectively. Based on the gene characteristics described below and observed tissue distribution (see Results, Tissue specificity of expression), we subsequently called them candidate *Aplysia californica *chemosensory receptors (AcCRs) subfamilies a to c.

#### Candidate *Aplysia californica *chemosensory receptors subfamily a

**Figure 2:**
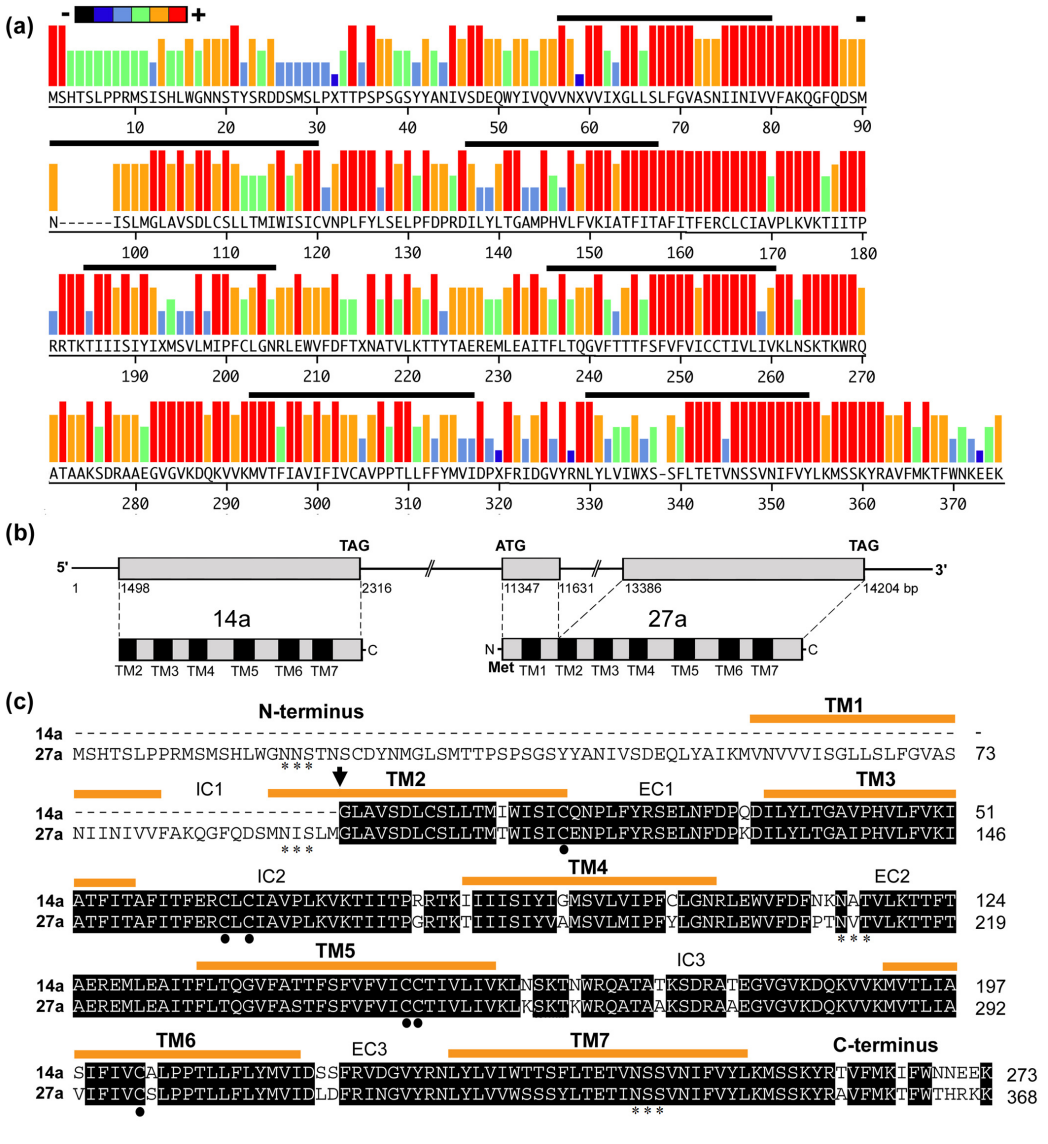
**Analysis of candidate *Aplysia californica *chemosensory receptors subfamily a**. **(a) **Conservation of predicted amino acid sequences for 10 full-length genes is displayed as a consensus strength as color-coded histogram. In this representation, the relative frequency with which an amino acid appears at a given position is reflected by the color, as depicted by the scale bar. The seven transmembrane domains (based on the HMMTOP version 2.0 program) are indicated by a solid bar above the sequence. **(b) **Schematic representation of the genome organization of clustered genes, partial *AcCR14a *and full-length *AcCR27a *(genomic contig AASC01105652), including predicted start (ATG) and stop codons (TAG) and intron/exon structure of AcCR27a leading to the mature multi-transmembrane protein. Met, methionine. **(c) **Comparative amino acid alignment of AcCR14a and AcCR27a. Identical amino acids are highlighted in black. Putative intracellular (IC), extracellular (EC), N-terminus and C-terminus domains are shown. Arrow indicates the intron/exon boundary; asterisks show potential N-linked glycosylation sites; black circles represent highly conserved cysteines.

A total of 28 genes encoding rhodopsin GPCR-like proteins were identified within the *Aplysia *genome that grouped into the single monophyletic AcCRa. Of these, 10 appear to encode 7-TM domain proteins that range in size from 355 to 368 amino acids (40.1 to 41.5 kDa). The remaining 18 genes encoded 6-TM domain proteins; however, we were unable to identify an initiator methionine suggesting that these represent partial length genes. Figure 2a is a comparative representation of the 7-TM proteins, showing conservation of predicted amino acid sequences for the gene repertoire. Overall, amino acid sequence identity among the subfamily ranges from 70% to 95%. These genes are most distinct from other subfamily sequences we identified due to the presence of a conserved intron between coding regions ISLM_95_/GLAV (based on *AcCR*27a, Figures 2b and 2c). This was later verified by reverse transcription-polymerase chain reaction (RT-PCR) cloning and sequencing (See Results: laser capture microdissection (LCM)/RT-PCR gene identification). Also, conservation of GLA/SV_96–99_, Y_120_, ITAFITF_150–156_, K_178_, G_198_, DRA/V_271–273_, MVT_287–289_, ET_336–337_, and NSSVNI_339–344 _are distinct this subfamily. Most variability is found within the N-terminal regions (also see alignments in the Additional file 2). Semi- and highly conserved cysteine residues are located at C_114_, C_159_, C_161_, C_247_, C_248 _and C_298_, while glycosylation sites can be found at N_18_NS, N_91_IS, N_210_A/VT and N_339_SS. Most share a signature motif with a FITAFITFERCLCIA amino acid sequence in the third transmembrane domain and second intracellular loop. We found that one genomic contig AASC01105652 contained two genes, *AcCR*14a and *AcCR*27a (Figure 2b). We predict that these are part of larger clusters that may become apparent upon completion of the full genomic assembly. Within the *Aplysia *genome (1.8 Gb), *AcCR*14a and *AcCR*27a are separated by 9031 bp, and are in the same transcriptional orientation. A comparative amino acid alignment of the partial AcCR14a and full-length AcCR27a proteins are shown in Figure 2c.

#### Candidate *Aplysia californica *chemosensory receptors subfamily b (AcCRb)

**Figure 3:**
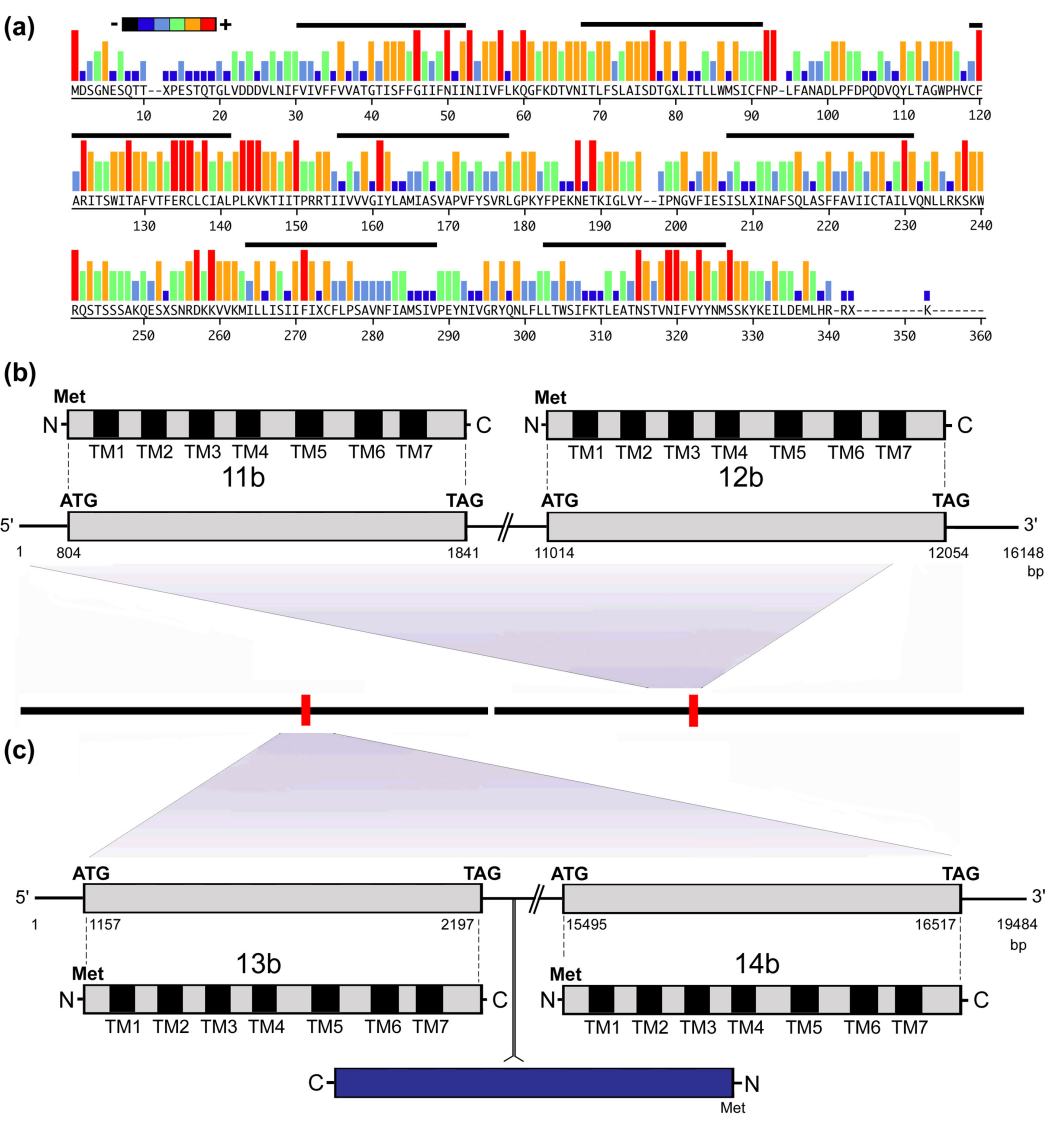
**Analysis of candidate *Aplysia californica *chemosensory receptors subfamily b**. **(a) **Conservation of predicted amino acid sequences for 38 full-length genes is displayed as a consensus strength as color-coded histogram. In this representation, the relative frequency with which an amino acid appears at a given position is reflected by the color, as depicted by the scale bar. The seven transmembrane domains (based on the HMMTOP version 2.0 program) are indicated by a solid bar above the sequence. **(b) **Schematic representation of the genome organization of clustered genes *AcCR11b *and *AcCR12b *(genomic contig AASC01159697), including predicted start (ATG) and stop codons (TAG). **(c) **Similar to (b) but clustered genes *AcCR13b *and *AcCR14b *(genomic contig AASC01064969). A predicted retrotransposon element is identified in the reverse orientation between the two gene sequences (see Figure S1 in Additional file 3). Met, methionine.

A total of 38 full-length intronless genes encoding predicted multi-transmembrane rhodopsin GPCR-like proteins were identified belonging to AcCRb. Sizes ranged from 319 to 364 amino acids in length (36.2 kDa to 40.8 kDa). Figure 3a is a comparative representation of these proteins, showing conservation of predicted amino acid sequence for the gene repertoire. Overall, amino acid sequence identity among the subfamily members ranges from 43% to 92%. Most variability is found at the N-terminal region and most conservation is within the predicted transmembrane 3 (also see alignments in Additional file 2). Most share a signature motif with a WITAFVTFERCLCIA amino acid sequence in the putative second intracellular loop. We found that at least some of the genes are clustered in the genome, including genes *AcCR*11b and *AcCR*12b (Figure 3b), as well as genes *AcCR*13b and *AcCR*14b (Figure 3c). Within the *Aplysia *genome, genes *AcCR*11b and *AcCR*12b are separated by 9173 bp while genes *AcCR*13b and *AcCR*14b are separated by 13297 bp, which also includes a putative non-long terminal repeat retrotransposon element in the reverse orientation (10532 to 9078 bp) (Figure S1 in Additional file 3). Amino acid identity between AcCR11b and AcCR12b, as well as AcCR13b and AcCR14b is high, 80.5% and 73.9% respectively (Figure S2 in Additional file 3). Conserved cysteines (based on AcCR11b) can be found at C_88_, C_133_, C_135_, C_221 _and C_269 _and N-linked glycosylation sites include N_5_ES, N_65_IT, N_184_KT, N_310_ST, and N_320_MS.

#### Candidate *Aplysia californica *chemosensory receptors subfamily c (AcCRc)

**Figure 4:**
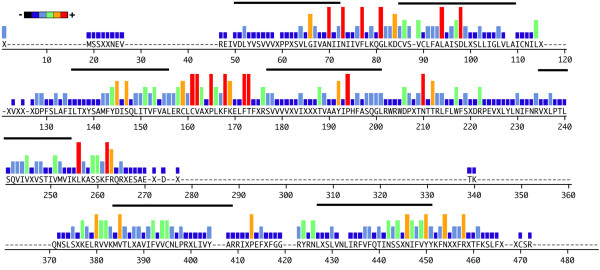
**Analysis of candidate *Aplysia californica *chemosensory receptors subfamily c**. Conservation of predicted amino acid sequences for 24 full-length genes is displayed as a consensus strength as color-coded histogram. In this representation, the relative frequency with which an amino acid appears at a given position is reflected by the color, as depicted by the scale bar. The seven transmembrane domains (based on the HMMTOP version 2.0 program) are indicated by a black bar above the sequence.

A total of 24 full-length genes containing uninterrupted coding regions were identified belonging to AcCRc. Sizes ranged from 324 to 433 amino acids in length (37.7 kDa to 49 kDa). Figure 4 is a comparative representation of these proteins, showing conservation of predicted amino acid sequence for the gene repertoire. Overall, amino acid sequence gene identity ranges from 19% to 91%. Most variability is found at the N-terminal region and within the proposed third intracellular domain, which carried length polymorphisms (also see alignments in Additional file 2). Conserved cysteines (based on AcCR2c) are located at C_136 _and C_138_, while semi- and highly conserved N-linked glycosylation sites are located at N_5_ET, N_16_IS, N_54_IT, N_187_TT, N_280_IS, N_331_TS.

### Tissue specificity of expression

**Figure 5:**
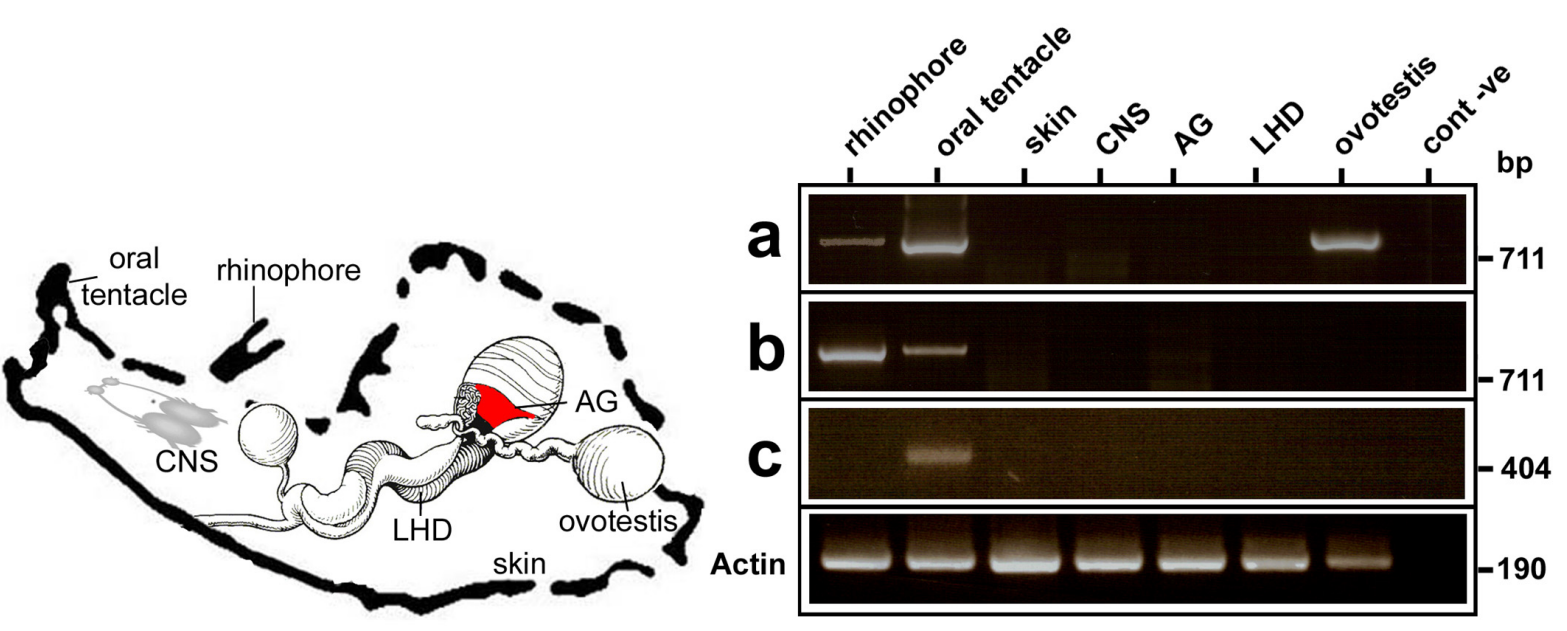
**Tissue-specific expression of candidate *Aplysia *chemoreceptors**. Schematic representation of *Aplysia californica *showing the location of selected sensory tissues (rhinophore, oral tentacle, skin), central nervous system (CNS) and reproductive organs (albumen gland (AG), ovotestis, large hermaphroditic duct (LHD)) used for RNA isolation and RT-PCR. Amplification products were 756 bp, 832 bp, 512 bp for AcCRa, AcCRb and AcCRc, respectively. The PCR control, using water instead of cDNA, was negative (cont -ve). Actin cDNA was amplified from each tissue preparation (224 bp).

We next studied the expression of subfamily genes in adult tissues using degenerate primers designed to conserved codons specific to each of the AcCR subfamilies. This approach was designed to detect if any members within the three subfamilies were expressed in the target tissues. Transcripts from AcCRa and AcCRb were identified in rhinophore, as well as the oral tentacle; AcCRa transcript was also present in the ovotestis (Figure 5). AcCRc transcripts were detected in the oral tentacle, indicating that each of the gene subfamilies are differentially expressed in the sensory tissues. No transcripts were detected in the skin, central nervous system, albumen gland or large hermaphroditic duct using the method described. We used the *Aplysia *housekeeping gene actin to demonstrate the integrity of each RT sample (224 bp). An alignment of deduced AcCRa amplicon sequences obtained from rhinophore, oral tentacle and ovotestis revealed that different members are present, which correspond most closely to genes *AcCR*5a (97%), *AcCR*19a (100%), and *AcCR*7a (90%), respectively (Figure S3 in Additional file 3). A comparative analysis of proteins encoded in AcCRb amplicons showed that the rhinophore and oral tentacle express a common candidate chemoreceptor gene, corresponding to the gene *AcCR*17b. Several point mutations, however, were present within the rhinophore transcript, which led to a premature stop codon (Figure S3 in Additional file 3). The AcCRc amplicon corresponded to the clone *AcCR*2c.

### Scanning electron microscopy and molecular identification of candidate chemoreceptors

**Figure 6:**
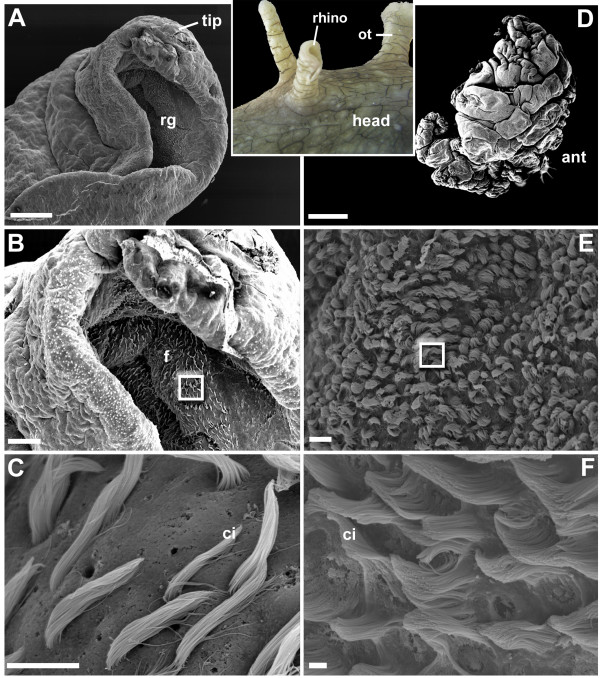
**Scanning electron micrograph analysis of two *Aplysia *chemosensory organs**. *Aplysia *species possess rhinophore (rhino) and oral tentacles (ot) to detect chemical stimuli in their marine environment. **(a to c) **Scanning electron microscopy (SEM) migrographs showing the surface of the *Aplysia *rhinophore. Scale bars: 300 μm, 100 μm and 10 μm, respectively. **(a) **A low-power SEM micrograph showing the rhinophore tip. rg, rhinophore groove; tip, rhinophore tip. **(b) **A medium-power SEM micrograph of the rhinophore tip showing the cilia-bearing epithelium within the rhinophore groove. f, folds. **(c) **Higher-power SEM micrograph of groove epithelium (boxed region in b) showing numerous bunched cilia extending from a common pore. Also evident are pores lacking obvious bunched cilia. ci, numerous long cilia. **(d to f) **SEM micrographs showing the surface of the *Aplysia *oral tentacle. Scale bars 1 mm, 10 μm and 1 μm, respectively. **(d) **A low-power SEM micrograph showing the oral tentacle. ant, anterior. **(e) **A medium-power SEM micrograph of the oral tentacle showing a mat of cilia-bearing epithelium. **(f) **Higher-power SEM micrograph of epithelium (boxed region in e) showing numerous bunched cilia extending from a common pore.

Based on results from tissue-specific expression experiments, we focused on the chemosensory organs. In reproductively mature *Aplysia *adults, the rhinophore (about 1 cm in length) is round and tapered from the base to the tip (Figure 6). Scanning electron microscopy (SEM) supports histological examination [27,28] and reveals that rhinophore grooves comprise folds of sensory epithelia bearing numerous cilia extending from a common pore (Figures 6a to 6c). The tip and outside surface of rhinophores are largely devoid of obvious cilia. This rhinophore groove epithelium was previously isolated by LCM and used to construct a cDNA library [12]. In a mature adult, the oral tentacle extends laterally and anteriorly from the ventral surface of the head, with an epithelium containing numerous bunched cilia (Figures 6d to 6f). Although ciliated regions were most common, the oral tentacle did contain regions of no obvious cilia. We next performed PCR with the aim to identify full-length candidate chemoreceptors from the rhinophore epithelium LCM library or prepared oral tentacle cDNA. Three clones were selected for further analysis. Subsequent protein sequence analyses using the Protein Families database of alignments and Interpro database [33] revealed that the deduced amino acid sequences have characteristics common to rhodopsin GPCRs (Figure S4 in Additional file 3).

#### AcCRa

**Figure 7:**
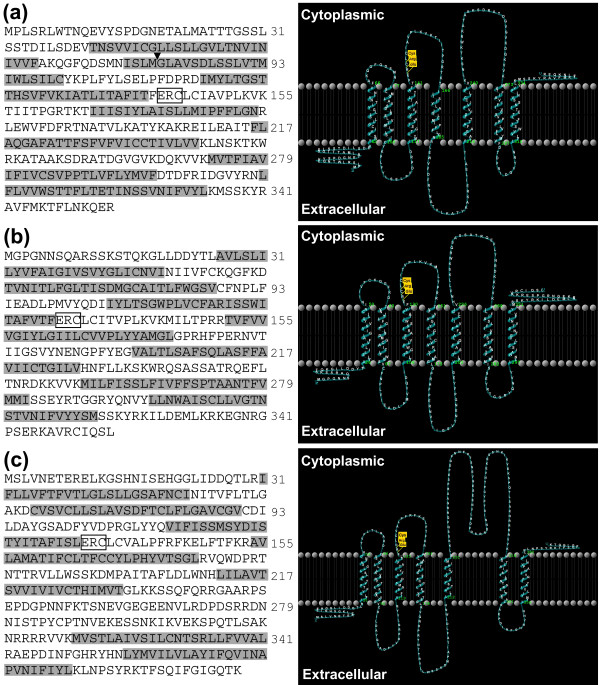
**Molecular identification of candidate *Aplysia californica *chemoreceptor genes**. Deduced amino acid sequence and schematic model of **(a) **AcCRa cDNA isolated from rhinophore laser capture microdissection (LCM) RNA (GenBank: EU935862), **(b) **AcCRb cDNA isolated from rhinophore LCM RNA (GenBank: EU808014) and, **(c) **AcCRc cDNA isolated from oral tentacle cDNA (GenBank: EU808013). Arrowhead, indicates position of intron/exon boundary and boxed grey areas indicate predicted transmembrane domains (based on HMMTOP version 2.0 program). Unfilled boxes show relative positions of the conserved motif Glu-Arg-Cys (ERC) at the proximal part of the cytoplasmic 2 domain.

PCR amplification of a rhinophore epithelium LCM library using degenerate primers that were selective for members of AcCRa sequences (primer combinations A1 to A5) generated amplification products of 757 bp. Several amplicons were successfully cloned and sequenced, revealing multiple partial-length AcCRa genes. Subsequently, the full length of one gene sequence was identified by 5'- and 3'-RACE, containing 1269 bp and encoding a protein of 354 amino acids (Figure 7a – corresponding most closely, 91%, to partial gene *AcCR*8a). The sequence data has been submitted to the GenBank database under accession number EU935862. It possesses three protein kinase C (PKC) phosphorylation sites (T_203_YK, S_256_DR, S_337_SK,) and four N-linked glycosylation sites (N_18_ET, N_76_IS, N_196_AT and N_325_SS). The intron/exon boundary exists between coding regions ISLM_80_/GLAV. Kyte-Doolittle hydropathy profiles indicate the existence of seven hydrophobic transmembrane segments that were composed of between 20 and 25 residues, connected by extracellular and cytoplasmic loops.

#### AcCRb

PCR amplification of a rhinophore epithelium LCM library using degenerate primers that were selective for AcCRb sequences (primer combinations B1 to B8) generated amplification products of 744 bp. Several amplicons were successfully cloned and sequenced, revealing multiple partial-length AcCRb genes. Subsequently, the full length of one gene sequence was identified by 5'- and 3'-RACE, containing 1483 bp and encoding a protein of 354 amino acids (Figure 7b – corresponding most closely, 99.4%, to gene *AcCR*29b). The sequence data has been submitted to the GenBank database under accession number EU808014. It possesses potentially six PKC phosphorylation sites (S_11_SK, T_15_QK, T_147_PR, T_249_NR, S_322_SK, S_343_ER) and four N-linked glycosylation sites (N_5_NS, N_65_IT, N_184_VT and N_310_ST) within the predicted N-terminal region and intracellular loop domains. Kyte-Doolittle hydropathy profiles of the deduced amino acid sequence showed that it contained seven hydrophobic transmembrane segments that were each composed of 25 residues.

#### AcCRc

AcCRc genes could not be PCR-amplified from a rhinophore LCM library. However, transcripts could be obtained from oral tentacle cDNA preparations (Figure 7c – corresponding most closely, 98%, to identified gene *AcCR*2c). PCR amplification of oral tentacle cDNAs using degenerate primers selective for AcCRc (gene combination C1) generated an amplification product of 824 bp. The amplicon was successfully cloned and sequenced. Subsequently, the full-length gene sequence was identified by 5'- and 3'-RACE, containing 1752 bp and encoding a protein of 398 amino acids. The sequence data has been submitted to the GenBank database under accession number EU808013. It possesses 10 PKC phosphorylation sites (T_7_ER, T_28_LR, T_150_FK, T_188_TR, S_195_SK, S_275_RR, S_295_NK, S_308_AK, T_332_SR, S_385_YR, a cAMP phosphorylation site (K_234_KSS) and six N-linked glycosylation sites (N_5_ET, N_16_IS, N_54_IT, N_187_TT, N_280_IS, N_329_TS) within the N-terminal region and intracellular loop domains. Kyte-Doolittle hydropathy profiles of the deduced amino acid sequence showed that it contained seven hydrophobic transmembrane segments that were composed of between 20 and 25 residues.

### Immunofluorescent localization of a candidate chemoreceptor

**Figure 8:**
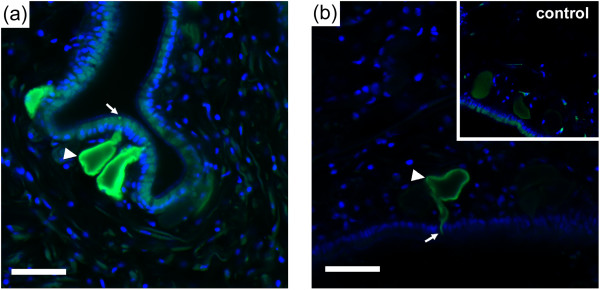
**Immunofluorescence localization of AcCR29b**. An antibody designed to the N-terminal region of AcCR29b was used to localize corresponding protein within epithelial cells of **(a) **rhinophore and **(b) **oral tentacle, shown in green. Arrowheads show immunopositive cell bodies and arrows point to processes exposed to the surface. Sections were counterstained with DAPI (blue) to show nuclear staining. A control in which the primary antibody was preabsorbed against its antigenic peptide showed greatly reduced staining at the same exposure (b, inset). Scale bars = 50 μm.

We complemented our RT-PCR gene expression study by analyzing the spatial distribution of AcCR29b protein within rhinophore and oral tentacle. Figures 8a and 8b shows representative sections of immunoreactivity within similar cell types located at the epithelial surface of rhinophore and oral tentacle, respectively. In both, antisera strongly label cell bodies and processes that extend to the surface, containing no apparent cilia. Rhinophore immunolabeling was most prominent in cells located in epithelia at the tip and outer surfaces, while not obvious within epithelium of the rhinophore groove at the magnification tested. Controls in which the primary antibody was preabsorbed against its antigenic peptide showed greatly reduced staining at the same exposure (Figure 8b, inset).

### Distribution of Gα_q_, Gα_i _and Gα_o _immunoreactivity in rhinophore

**Figure 9:**
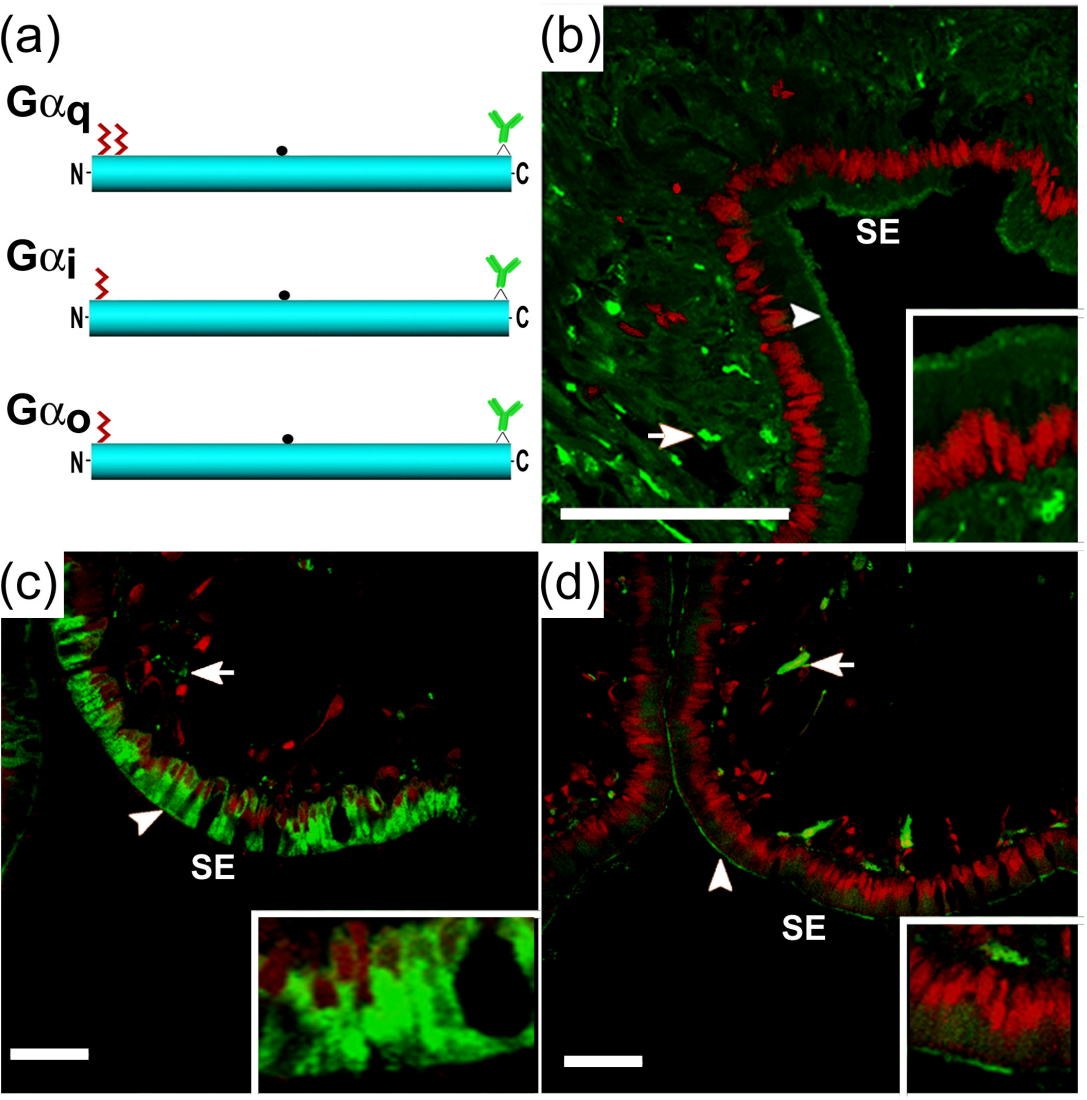
**Analysis of *Aplysia *Gα_q_, Gα_i_, and Gα_o _proteins in rhinophore**. **(a) **Schematic diagrams of Gα_q _(GenBank: DQ397515), Gα_i _(GenBank: DQ656111) and Gα_o _(GenBank: DQ656112) showing the location of N-terminal cysteines that may be sites for palmitoylation (wavy lines), a putative cholera toxin ADP-ribosylation site (●), and the binding site of Gα-specific antibodies used for immunolocalization analyses. **(b to d) **Immunofluorescent (green) localization of Gα_q _**(b)**, Gα_i _**(c) **and Gα_o _**(d) **in the rhinophore sensory epithelium (SE), including presumptive sensory neuron cell fibers (arrows) and outer epithelia (arrowheads). Sections were counterstained with propidium iodide (red). Scale bars = 100 μm. Higher magnifications are shown in insets.

Sections were taken from the sites of pheromone detection, the rhinophore. *Aplysia *G proteins encoded by previously isolated transcripts from rhinophore sensory epithelium [29] are shown schematically in Figure 9a. Commercial antibodies used for this study were directed to the C-terminus which shares 100% identity with *Aplysia *Gα proteins. Immunofluorescence studies confirmed the immunoblot expression results [29] and demonstrated localization of immunoreactive *Aplysia *Gα_q _in rhinophore sections. Numerous Gα_q _immunoreactive fibers were observed proximal to the rhinophore epithelium and within the distal layer (Figure 9b); Gα_q_immunoreactivity appeared to be present in the outer sensory surface, consistent with a potential role in pheromone signal transduction. Gα_i_-labeled cells were identified throughout rhinophore sections, where immunoreactivity was particularly concentrated in the distal regions of sensory epithelia. Inspection of these sections at high magnification revealed that the majority of cells in the epithelium were labeled, including cells presumed to be supporting cells and sensory neurons (Figure 9c). Immunoreactive fibers could also be found spreading into the cortex of the rhinophore, although this was less prominent. In contrast, immunoreactive *Aplysia *Gα_o _had a more restricted distribution, in that each section contained several immunoreactive fibers that were observed primarily within presumptive olfactory neuron dendrites (Figure 9d); fewer immunoreactive presumptive sensory neuron cell bodies were observed. No immunoreactivity was observed when primary antibody to Gα_q_, Gα_i _or Gα_o _was omitted (data not shown).

## Discussion

In this study, we provide an important step towards understanding the molecular and cellular basis of chemosensory recognition in molluscs. Mining of the incomplete *A. californica *genome revealed a novel and diverse set of genes encoding 90 (72 considered full-length intact) rhodopsin GPCR-like proteins that are likely to mediate chemosensory responses in *Aplysia*. To initially identify genes encoding multi-transmembrane GPCR-like proteins that may play a role in chemosensory detection, a number of assumptions were made that have proved important for the successful isolation of chemoreceptors in other metazoans. First, receptor genes would encode 7-TM domains and be clustered in the genome. Second, receptors would be relatively rapidly evolving and thus have limited amino acid sequence identity to members of known conserved GPCR families. Finally, receptors would be encoded by unique families of related genes, as has been observed in a range of other bilaterians [5,15,34]. Phylogenetic analysis of the identified *Aplysia *genes revealed the existence of three monophyletic subfamilies, which we have named candidate *Aplysia californica *chemosensory receptor (AcCR) subfamilies a to c; their primary features are summarized in Table 1. The gene expansion observed could provide the diversity of receptors required to enable the animal to recognize diverse water-soluble molecules, as well as complex pheromone blends during the coordination of attraction and reproduction.

**Table 1:** Summary of candidate *Aplysia *chemoreceptor genes selected by genome mining

Subfamily	Number of intact genes	Size range (aa's)	Predicted TM domains	Intron number	Sites of expression**
a	10*	355–368	7	1	rhino, ot, ovo
a	38	319–364	7	0	rhino, ot
c	24	324–433	7	0	ot

* Not including those considered partial-length (non seven transmembrane domain).

** Based on degenerate reverse transcription-polymerase chain reaction strategy outlined in this study (Methods)

rhino, rhinophore; ot, oral tentacle; ovo, ovotestis.

Consistent with known chemosensory receptors (for example, insect, rodent, fish), all selected genes that were considered full length encoded 7-TM regions and semi-conserved glycosylation sites, as well as several common cysteine residues and amino acid sequence motifs. Conserved amino acids and post-translational modifications would likely contribute to the correct folding and functioning within the plasma membrane so that they may bind chemical stimuli and couple to appropriate secondary signaling molecules. For example, Katada and colleagues [35] demonstrated that in rodents, N-terminal glycosylation is critical for proper targeting of ORs to the plasma membrane. While proteins encoded by AcCRa and AcCRb genes share notably high sequence identity, comparative analysis within AcCRc shows that they share as little as 19% amino acid identity. As a consequence, there are very few defining sequence motifs which are retained throughout. However, it is not uncommon for chemoreceptors, and in particular gustatory receptors, to be divergent; similarity between most insect and mammalian gustatory receptor pairs is only 15% and 25% or less at the amino acid sequence level, respectively [10,36].

Of the major GPCR superfamily groups, the identified *Aplysia *genes categorize most closely to the rhodopsin GPCR family (based on Interpro database [33]). As is the case with many other rhodopsin family GPCRs, these genes largely lack introns. Moreover, all encoded proteins have a short N-terminus and a highly conserved arginine (R) residue located at the cytosolic end of the third transmembrane domain. This residue is typically associated with the DRY (Asp-Arg-Tyr) motif, crucial for controlling agonist-dependent receptor activation. Of the three residues constituting DRY, arginine is the most conserved residue, and appears to be essential for forming intramolecular interactions that constrain receptors in either the inactive or activated conformation [37]. Consistent with this, receptors lacking the arginine side chain fail to activate G-protein signaling [38,39]. In the novel *Aplysia *proteins, however, this has been replaced by an ERC motif, a feature also observed in the human prostaglandin F2α receptor and most other prostanoid receptors [40]. Studies show that substitution of the glutamic acid to a threonine residue leads to full constitutive activation and implicates the region in agonist-dependent G-protein coupling control [40]. We predict that this may also be essential to receptor activation in the identified *Aplysia *receptors.

The canonical model of GPCR activation is via an interaction with intracellular heterotrimeric G-protein signaling components. The genes identified in this study show little amino acid identity to GPCRs found in the Metazoa and we have not shown that they directly interact with G-proteins. Despite this, our study indicates that Gα proteins are present in the rhinophore sensory epithelium, possibly in close association with transmembrane GPCRs. The presence of sensory tissue G-protein immunoreactivity adds further support to studies of other marine invertebrate olfactory systems implicating G-proteins in sensory transduction [21,41]. Moreover, the existence of multiple G-type proteins in sensory epithelium suggests that multiple signal transduction pathways may be activated following ligand stimulus. In squid, for example, the pattern of immunolabeling implies that a G protein coupled to a PLC pathway (Gα_q _and Gα_o_) may be present in similar cells as those coupled to a cAMP pathway (Gα_i_). As suggested by Mobley and colleagues [21], overlapping G-protein pathways could facilitate discrimination between odorants detected by the same neuron. This contrasts rodent models where the role of G proteins in olfactory transmembrane signaling at the dendrites has been studied extensively. Researchers have demonstrated spatially restricted patterns of expression of respective G proteins [42-44]. Gαo and Gαi2 are highly expressed by separate subsets of neurons that are located in different regions of the vomeronasal neuroepithelium [44].

Of particular relevance to *Aplysia *chemosensory studies is the rhinophore epithelium, where water-borne molecules such as pheromones presumably bind and initiate activation of pheromone-receptive neurons. In the rhinophore groove, receptor cells with a suspected chemosensory role have been described previously in molluscs [26-28] and their presence was further supported by our SEM analysis. The rhinophore groove ciliary aggregations are likely necessary in the separation and circulation of fluid throughout the groove, and may also be directly involved in detection of external chemical stimuli. It is from this precise location that we isolated cDNAs encoding identified novel GPCR-like proteins. Their presence raised the question as to whether their expression was specific to sensory tissues. Subsequently, representatives of each subfamily were found to be restricted to or differentially expressed in the rhinophore, oral tentacles and ovotestis, suggesting that they encode functional receptors and that these olfactory organs sense different chemicals. In the rhinophore, spatial expression of cells immunoreactive to candidate chemoreceptor AcCR29b was most prominent in the tip and outer epithelium, peripheral to the groove. Chemosensory detection could likely benefit from this broad distribution, whereby stimulation may activate sensory fibers that extend to higher brain centers. Although this finding clearly indicates a sensory role, a more extensive study of *Aplysia *sensory organs at higher magnification is required to delineate the precise distribution of this receptor, as well as other receptor subfamily members.

Interestingly, we found gene expression within the ovotestis, and our preliminary analysis of various *Aplysia *neuronal EST databases indicate that a relatively small fraction of these genes may be expressed in the central nervous system. Deep sequencing of neuronal transcripts has resulted in identification of tags for 13 different genes in the central ganglia of *A. californica *(that is, *AcCR*1a, *AcCR*5a, *AcCR*15a, *AcCR*32b, *AcCR*5c, *AcCR*9c, *AcCR*11c, *AcCR*15c, *AcCR*16c and *AcCR*20c, see Additional file 1) as well as several orphan receptors similar to vertebrate ghrelin and histamine receptors (L Moroz, unpublished). Some of those are associated with centrally located sensory neurons that send neuronal processes to the periphery and therefore may be involved in chemoreception. Other neuronal cell types were previously described as motorneurons. Taken together, these findings imply that external chemical detection may not be their sole function, which is consistent with that described of chemoreceptors in mammalian olfactory bulb [45], cardiac muscle [46] and vertebrate germ tissues [47-49]. The functional significance of such expression is currently unknown. We speculate that there may be a role for *Aplysia *chemoreceptors in oocyte recognition, possibly because both are activated by identical or structurally similar hormones and pheromones. In addition to the exocrine albumen gland, the *Aplysia *pheromone seductin is known to be expressed in ovotestis tissue [24].

It will be of interest to perform a comparative gene analysis to determine whether *A. californica *candidate chemoreceptors are also present in other *Aplysia *species. This finding would suggest that these genes are highly similar throughout *Aplysia *species and strongly imply a selective pressure for conservation. We have already established that several *Aplysia *species share a comparable attraction pheromone blend [4,23], and therefore cognate receptor binding sites are likely to be similar. The identification of ligands for chemosensory receptors is often problematic and therefore it is an advantage that we have these attraction pheromones for use in future functional studies to definitively link identified genes to chemosensory detection. As these are the first such novel GPCRs identified in molluscs, it will also be of interest to see if analogous receptors are found in evolutionarily distant molluscs. However, apart from the highly conserved insect receptor Or83b [50], it is generally accepted that chemoreceptors seem to be very divergent with little sequence conservation within and across orders [51,52].

Our analysis of the *Aplysia *genome noted that AcCRa genes as well as various AcCRb genes are clustered, a common feature of fast-evolving genes such as chemoreceptor genes [15,53-55]. We also found that, although the assembly of the genome used was incomplete, some genes contain mutations that introduce stop codons to encode truncated proteins, one of which appears to be expressed in the *A. californica *rhinophore. Hominoids, in particular, are known to possess a high pseudogene content (50%) among their ORs, whereas only 20% of OR genes are pseudogenes in the mouse [34] and less in the *Drosophila melanogaster *genome [56]. Upon genome completion, a more comprehensive analysis of GPCR gene families in *Aplysia *will be necessary to determine the precise pseudogene number. Indeed, some of the pseudogenes identified may in fact be 'flatliners', that is, genes whose functional versus pseudogene status is unclear [57]. As demonstrated in *C. elegans*, many of these genes have apparently functional alleles in one or more wild isolates and therefore are not pseudogenes. Evidence for this has also been shown for some *Drosophila *ORs [58] and gustatory receptors [59], as well as *Anopheles gambiae *gustatory receptors [51]. Although pseudogenes are generally accepted as nonfunctional and therefore not transcribed, occasionally it has been shown that such pseudogenes can be transcribed [60]; however, there is no evidence of the functional relevance.

## Conclusion

*Aplysia *is an excellent model animal for studying the molecular mechanism of chemical communication in the marine environment. In this study we have isolated a novel group of genes encoding multi-transmembrane rhodopsin GPCR-like proteins that show expression in chemosensory tissues. This expression pattern and observed genomic clustering provide strong evidence that these have arisen via gene duplication and may be used to discriminate the large diversity of water-soluble molecules. The expression of some of these in rhinophore suggests that they are excellent candidates to be involved in pheromone detection. Further knowledge of the receptor gene genome organization, characterization of their developmental and spatial expression profile, secondary signaling and their evolutionary relationship to other molluscan species would be the next significant steps towards defining the logic behind how chemical communication in molluscs, and potentially other marine animals, operates.

## Methods

### Database mining for multi-transmembrane rhodopsin G-protein coupled receptor-like genes

*A. californica *genome contig sequences were procured from the NCBI trace database [61]. This was a preliminary genome assembly from 2× coverage of the genome by the Broad Institute at MIT [62]. An iterative tBLASTn strategy was adopted to identify multi-transmembrane rhodopsin GPCR-like genes in the *Aplysia *genome. Selection criteria included that receptors would be encoded by a family of related genes; at least some receptor genes would be clustered at the same genetic loci; receptors would have limited amino acid identity to members of known GPCR superfamilies; and a full-length coding region would encode multiple transmembrane domains. A search was initially performed using molluscan GPCR protein sequences already submitted to GenBank, as queries; a non-stringent expectation value cutoff of 1e-4 was employed. During this search we retrieved two sequences on contig AASC01105652 with genes encoding hydrophobic multi-transmembrane domains with no significant amino acid identity to other proteins. Putative *Aplysia *transmembrane receptors were in turn employed in searches to find more genes in an iterative tBLASTn process. A candidate rhodopsin GPCR-like gene having a complete open reading frame (methionine, 7-TM domains, three extracellular domains, three intracellular domains, and a stop codon) was considered intact and probably functional. To be conservative, genes that were >98% identical in amino acid sequence were considered allelic variants. As the *Aplysia *genome has not yet been fully assembled and consists of only contigs, this method does not identify splice-variants or provide a comprehensive analysis of gene clustering. Pseudogenes were identified by premature stop codons and we have arbitrarily chosen to disregard those genes that encode less than six transmembrane domains. Computer analyses of sequences were performed using BLAST and CLUSTALW for nucleotide alignment. Transmembrane helix domain and topology of predicted receptors was performed using HMMTOP version 2.0 program; [63]. Hydrophilicity plots were generated using the TMHMM program at [64]. In order to categorize identified genes, we used [65] and the Interpro database [33].

### Phylogenetic and gene analysis

Selection of outgroup sequences was performed by GenBank tBLASTn searches. The approach provided homologous counterparts from *C. elegans *for the *Aplysia *rhodopsin GPCR-like genes representing subfamilies a and b. We did not find any significant counterparts for subfamily c. The sequences were aligned with t-coffee [66] under default settings. Alignments have subsequently been improved by eye. Character positions, which could not be aligned unambiguously, were not considered for the phylogenetic analyses in order to avoid conflicting phylogenetic signal. The inclusion of outgroup sequences of *C. elegans *and *Strongylocentrotus purpuratus *resulted in a large proportion of only ambiguously alignable positions and consequently required deletion of a relatively high number of characters. Therefore we performed two separate analyses: first, we aligned and analyzed the ingroup sequences only in order to reconstruct their evolution under a maximal number of informative characters. Second, we aligned and analyzed the dataset with the non-*Aplysia *sequences included in order to infer the polarity to the phylogeny.

Phylogenetic analyses were conducted on a multi-processor Linux-Cluster under the likelihood criterion using RAxML v. 7.0 [67] for Maximum Likelihood and MrBayes v. 3.1.2 [68] for the Bayesian Inference. MrBayes analyses were performed in two runs of eight MCMCMC chains and under the GTR+G+I Model [69]. Chains ran for 10,000,000 generations or were stopped when the standard deviation of split frequencies between both runs fell below 0.01. RAxML bootstrap analyses on 1,000 replicates have been performed under the PROTMIX algorithm with the WAG amino acid substitution model [70]. In PROTMIX the tree inference is performed under the PROTCAT model followed by the final tree evaluation under the PROTGAMMA model in order to obtain stable likelihood values (see the RAxML manual for further details).

### Animal and sample preparation

Adult *A. californica *(100 to 500 g) were obtained from Marine Research and Educational Products (Escondido, CA, USA). Animals were anesthetized in isotonic MgCl_2 _(337 mM) equivalent to 50% of their weight, relevant tissues dissected out and either (1) embedded in optimal cutting temperature (OCT) compound for LCM or sectioning, (2) snap frozen in liquid nitrogen for RNA and protein isolation, or (3) prepared for SEM.

### Reverse transcription-polymerase chain reaction

Total RNA was extracted from rhinophore, oral tentacle, skin, pooled central nervous system, albumen gland, large hermaphroditic duct, and ovotestis tissues of *A. californica *using a Tripure Isolation Reagent. Any contaminating genomic DNA was removed by treatment with DNase I followed by lithium chloride/ethanol precipitation. First-strand cDNA synthesis was performed in a 20 μl reverse transcription mixture containing oligo d(T)_12–18 _and 200 U Superscript III RNase H^- ^reverse transcriptase, following the manufacturer's instructions. PCR was performed using 1 μl of prepared cDNA using subfamily-specific primers (see Table S1 of Additional file 3: primer combinations A3, B3 and C1). Each reaction was performed in a final concentration of 1× PCR Buffer, 1.5 mM MgCl_2_, 200 μM of each dNTP, 0.5 μM of sense and antisense primer, 1.25 units of Red *Taq *polymerase and ddH_2_O. Negative controls contained no template cDNA. PCR using actin-specific primers (sense, 5'-GCTTCACCACCACTGCCGAGAG-3' and antisense, 5'-ACCAGCAGATTCCATACCCAGG-3') were used to ensure the integrity of each tissue cDNA sample. Reactions were heated at 94°C for 2 min and amplified for 36 cycles (94°C, 60 s; 50 to 55°C, 30 s; 72°C, 60 s). Following PCR, 15 μl of reaction mix was fractionated on 2% agarose gels and visualized by ethidium bromide staining. Based on the primer design, the expected amplification sizes were 756 bp (AcCRa), 832 bp (AcCRb), 512 bp (AcCRc) and 224 bp (actin). PCR products were cloned into pGEM-T vector and sequenced.

### Scanning electron microscopy of rhinophore and oral tentacle

Adult *Aplysia *rhinophore and oral tentacle were fixed with 2.5% glutaraldehyde in phosphate buffer (pH 7.2 to 7.4) for 3 days at 4°C. Secondary fixation was in 1% OsO_4 _(osmium tetroxide) in 0.1M sodium cacodylate. This material was dehydrated in a graded series of ethanol (20% to 100%). The samples were dried using hexamethyldisilazane and platinum-coated using Eiko IB-5 Sputter Coater. The specimens were viewed using a Jeol 6300 Field Emission Scanning Electron Microscope.

### Laser capture microdissection and molecular identification of candidate *A. californica *chemosensory receptors

The location of the rhinophore groove, glomeruli underlying the sensory epithelium, and rhinophore ganglia in *Aplysia *have been described previously [26-28]. To examine whether selected genes are expressed in rhinophore sensory epithelial cells, a combination of LCM, total RNA isolation, library construction and RT-PCR were performed. Rhinophore tissue that had been embedded in OCT compound was sectioned (10 μm) onto slides and dehydrated. LCM was performed using a PixCell II laser capture microscope with an infrared diode laser (Arcturus Engineering Inc., Mountain View, CA, USA) and a laser spot size of 15 μm. Cells were marked, captured on CapSure HS caps (Arcturus), and total RNA was isolated using the Picopure Isolation Kit (Arcturus) including DNase I incubation. RNA quality was determined by measurement of absorbance ratio at 260 nm/280 nm.

First-strand cDNA was synthesized according to the SMART cDNA Library Construction Kit protocol (Clontech, Palo Alto, CA, USA), with the minor modification of incorporated *Eco*RI restriction sites during cDNA synthesis (5'-AAGCAGTGGTATCAACGCAGAGTGAATTCACGCGGG-3' and 5'-AAGCAGTGGTATCAACGCAGAGTGAATTCT_30_VN-3'). Amplification of cDNA was performed by PCR using 5' and 3' PCR Primer mix (5'-CTAATACGACTCACTATAGGGCAAGCAGTGGTATCAACGCAGAGT-3' and 5'-AAGCAGTGGTATCAACGCAGAGTGAATTCT_30_VN-3'); samples were heated at 95°C for 1 min and amplified for 30 cycles (95°C, 1 min; 50°C, 30 s; 68°C, 4 min). Reaction volumes of 50 μl were treated with 2 μl of proteinase K (20 μg/μl) at 45°C for 20 min, and PCR products were precipitated. Dried samples were resuspended in 80 μl of deionized water. *Eco*RI digestion was performed and size fractionation was achieved using a CHROMA SPIN-400 column (Stratagene, La Jolla, CA, USA). Products were purified by precipitation and the dried pellet resuspended in 7 μl deionized water. *Eco*RI cDNA was cloned into the *Eco*RI sites of Lambda ZAP II vector, and the cDNA library (complexity 1 × 10^6^) amplified once.

The sequences of oligonucleotide primers (Sigma-Genosys, Australia) used for library PCR are located in Table S1 of Additional file 3. For AcCRa and AcCRb genes, PCR was performed using degenerate sense and antisense primer combinations A1 to A5 (AcCRa) and B1 to B8 (AcCRb). Each reaction was performed using Red *Taq *polymerase (Sigma) following the manufacturer's instructions. Samples were heated at 94°C for 3 min and amplified for 36 cycles (94°C, 60 s; 45°C, 30 s; 72°C, 60 s), followed by a 7-min extension at 72°C. PCR products were cloned into the TA vector pGEM-T (Promega) and sequenced as previously described [23]. To obtain 5' and 3' sequences, PCR was performed using gene-specific primers. For 3'-RACE, gene-specific sense primers (A3' and B3') were used in combination with vector primer T3. For 5'-RACE, gene-specific antisense primers (A5' and B5') were used in combination with vector primer T7. Samples were heated at 94°C for 2 min and amplified for 36 cycles (94°C, 60 s; 50°C, 30 s; 72°C, 60 s), followed by a 7-min extension at 72°C. PCR products were cloned into pGEM-T vector and sequenced.

### Molecular identification of candidate Aplysia chemosensory receptors within subfamily c

Total RNA was extracted from oral tentacle tissue of *A. californica *using a Tripure Isolation Reagent (Roche), and any contaminating genomic DNA was removed by treatment with DNase I (Invitrogen). First strand cDNA synthesis was performed using 1 μg of total RNA in a 20 μl reverse transcription mixture containing oligo d(T)_12–18 _and 200 U Superscript™ III RNase H^- ^reverse transcriptase, following the manufacturer's instructions. The sequences of oligonucleotide primers used for PCR are located in Table S1 of Additional file 3. PCR was performed using degenerate sense and antisense primer combinations C1-C3. 3'- and 5'-RACE was performed with the SMART RACE amplification kit (BD Biosciences) and using gene-specific primers sense and antisense primers (C3' and C5'). Samples were heated at 94°C for 2 min and amplified for 36 cycles (94°C, 60 s; 50°C, 30 s; 72°C, 60 s), followed by a 7-min extension at 72°C. PCR products were cloned into a pGEM-T vector and sequenced.

### Immunohistochemical localization

A rabbit polyclonal antibody was generated to the N-terminal region of candidate chemoreceptor 29b, corresponding to N_6_SQARSSKSTQKGL (GenScript Corporation). This region was chosen due to its lack of significant amino acid identity to other receptors. Details of the immunohistochemical protocol have been described [23,24]. Briefly, tissue cryostat sections of rhinophore were incubated overnight at 4°C in either affinity-purified 29b antibody (0.6 mg/ml, 1:500), Gα_q_, Gα_i _or Gα_o _antisera (Chemicon, 1:500 dilution), rinsed in phosphate buffered saline (PBS), incubated in fluorescein isothiocyanate (FITC)-conjugated goat anti-rabbit Ig (Sigma-Aldrich, St. Louis, MO, USA) for 1 h at 22°C, rinsed in PBS, and then mounted in FITC mounting media (90% glycerol/100 mM Tris pH 8.0). Preparations were examined using an Olympus FluoView confocal microscope (Leeds Precision Instruments, Inc., Minneapolis, USA), and the images captured on a spot-cooled charged coupled device camera. Sections were counterstained with 4',6-diamidino-2-phenylindole or propidium iodide at 1 μg/ml in water. As a control, the primary antiserum was replaced with no primary antibody. For 29b, a control also included using the primary antibody that had been preabsorbed against its antigenic peptide (20 μg/ml).

## Abbreviations

7-TM: seven transmembrane; AcCR: *Aplysia californica *chemosensory receptor; BLAST: basic local alignment search tool; EST: expressed sequence tag; FITC: fluorescein isothiocyanate; GPCR: G-protein coupled receptor; LCM: laser capture microdissection; OCT: optimal cutting temperature; PBS: phosphate-buffered saline; PLC: phospholipase C; RT-PCR: reverse transcription-polymerase chain reaction; OR: olfactory receptor.

## Authors' contributions

SFC, DE, GTN and BMD designed research. SFC and DE performed research. LLM and BMD analyzed data. SFC, ZZ, CC, GTN and BMD wrote the paper. All authors read and approved the final manuscript.

## Supplementary Material

Additional file 1 ***A. californica *rhodopsin G-protein coupled receptor-like genes**. This file summarizes details for all identified rhodopsin G-protein coupled receptor-like genes. Click here for file

Additional file 2 **Candidate *A. californica *chemosensory receptor alignments**. The complete comparative amino acid alignments within AcCRa-c proteins. Click here for file

Additional file 3 **Analysis of contig sequences and candidate chemosensory receptors**. Contains Figure **S1**, which shows the encoded protein sequence for a putative *Aplysia *reverse transcriptase (RNA-dependent DNA polymerase)-like gene; Figure **S2**, a comparative amino acid analysis for clustered AcCRb genes 11b/12b and 13b/14b; Figure **S3**, Comparative amino acid alignments of translated reverse transcription-polymerase chain reaction (RT-PCR) amplicons; Figure **S4**, Molecular identification of *Aplysia californica *chemosensory genes; and Table **S1**, a list of primers used for RT-PCR, PCR and gene cloning. Click here for file
