# Supplementary material for: Candidate chemoreceptor subfamilies differentially expressed in the chemosensory organs of the mollusc Aplysia
Source: BMC Biol. 2009 Jun 4;7:28. doi: 10.1186/1741-7007-7-28 (PMC2700072; doi:10.1186/1741-7007-7-28)
Supplement: Additional file 2 — Candidate A. californica chemosensory receptor alignments. The complete comparative amino acid alignments within AcCRa-c proteins. [file 1741-7007-7-28-S2.pdf]

Multiple files are bound together in this PDF Package.

Adobe recommends using Adobe Reader or Adobe Acrobat version 8 or later to work with documents contained within a PDF Package. By updating to the latest version, you'll enjoy the following benefits:

- Efficient, integrated PDF viewing
- Easy printing
- Quick searches

Don't have the latest version of Adobe Reader?

Click here to download the latest version of Adobe Reader

If you already have Adobe Reader 8,
click a file in this PDF Package to view it.
