# Supplementary material for: Candidate chemoreceptor subfamilies differentially expressed in the chemosensory organs of the mollusc Aplysia
Source: BMC Biol. 2009 Jun 4;7:28. doi: 10.1186/1741-7007-7-28 (PMC2700072; doi:10.1186/1741-7007-7-28)
Supplement: Additional file 3 — Analysis of contig sequences and candidate chemosensory receptors. Contains Figure S1, which shows the encoded protein sequence for a putative Aplysia reverse transcriptase (RNA-dependent DNA polymerase)-like gene; Figure S2, a comparative amino acid analysis for clustered AcCRb genes 11b/12b and 13b/14b; Figure S3, Comparative amino acid alignments of translated reverse transcription-polymerase chain reaction (RT-PCR) amplicons; Figure S4, Molecular identification of Aplysia californica chemosensory genes; and Table S1, a list of primers used for RT-PCR, PCR and gene cloning. [file 1741-7007-7-28-S3.pdf]

Figure S1. Putative retrotransposon element gene. **(a)** A predicted full-length gene encoding a 482 amino acid protein belonging to the RT-like superfamily was identified between subfamily b genes in the reverse orientation. It has most similarity with RT non-long terminal repeat retrotransposons. **(b)** Comparative amino acid alignment of *Aplysia* RT-like protein with reverse transcriptase proteins. # denotes putative active sites, including nucleic acid binding and putative NTP binding sites. GenBank accession nos. 4185148, putative reverse transcriptase (*Arabidopsis thaliana*); 903661, reverse transcriptase (*Ascaris lumbricoides*); 120551: retrovirus-related Pol polyprotein from type-2 retrotransposable element R2DM (*Drosophila*); 33301425: Retrotransposon (*Girardia tigrina*).

Figure S2. Comparative amino acid analysis of *A.californica* rhodopsin GPCR-like subfamily b. **(a)** Deduced amino acid alignment of genes 11b and 12b, and **(b)** 13b and 14b. Identical amino acids are highlighted in black. Putative intracellular (IC), extracellular (EC), N-terminus and C-terminus domains are shown.

Figure S3. Comparative amino acid alignments of translated RT-PCR amplicons. **(a)** Comparative amino acid alignment of AcCRa obtained from rhinophore (rhino), oral tentacle (ot) and ovotestis (ovo). **(b)** Comparative amino acid alignment of AcCRb obtained from rhinophore (rhino) and oral tentacle (ot). Identical amino acids are shaded in black. The seven transmembrane (TM) domains are indicated by an orange bar above the sequence.

Figure S4. Molecular identification of *Aplysia californica* rhodopsin GPCR-like genes. **(a)** Nucleotide sequence (1269 bp) and deduced amino acid sequence (354 aa) of a AcCRa cDNA isolated from rhinophore LCM RNA (GenBank: EU935862). **(b)** Nucleotide sequence (1483 bp) and deduced amino acid sequence (354 aa) of a AcCRb cDNA isolated from rhinophore LCM RNA (GenBank: EU808013). **(c)** Nucleotide sequence (1752 bp) and deduced amino acid sequence (398 aa) of a AcCRc cDNA isolated from oral tentacle cDNA (GenBank: EU808014). Boxed blue areas indicate predicted transmembrane domains. Underlined amino acids represent conserved N-linked glycosylation sites and boxes show location of cysteines. Grey highlight in **(c)** represents hypervariable region. Kyte-Doolittle hydropathy profile of each deduced multi-transmembrane receptor sequence is shown below each sequence. The approximate positions of the seven transmembrane domains are indicated above the hybropathy plot.

Table S1. PCR primers. Sequences of PCR primers used for degenerate PCR, 3′- and 5′- RACE and tissue-specific RT-PCR analysis. A1-A5, AcCRa primer sets; B1-B8, AcCRb primer sets; C1-C3, AcCRc primer sets; A3′, B3′ and C3′, 3′-RACE primer sets; A5′, B5′ and C5′, 5′-RACE primer sets.

Figure S1.

(a)

```
MPPHLEDVCCEEQDTILCLVGNIKIFLFNRTVLVYESLYIPQVLVYDAIVVPIWKKKGSKKDCNTYRGISLLS
HVGKMYAKILERRTRAKTEHLLSDAQFGFRKGRGCTDAIFALRQLCERALEYDKDLHLVFVDQEKAFDRVNRN
KLWKILEQYDIKGQLLDKIRAIYANSRSAVRTTSGTSDWFPVTSGVRQGCNLSPLLFVIYMDQITKEANPDPE
SLNELMFADDLAIINNKTQLQEHINQLNASCEKYDMKISISKTEVMTISRRPGKVDININGSQLKQSREFKYL
GSIFRENGKLDREIETRCQRANAISYQLGPLLKHPNIPMSTKVKLINAIFLPTLTYQCQTWTLTKALERKLVT
CEMKCLRRAVNKTRRDKIKNEVIRDTVGATPALPYIEKQRVKWFEHLTRMPSNHPALRAYNIKYSGWRRGRPR
RRWSESVADTLKDHGMSLLQATRLAADRHLCLPATPQGTSGRKK
```

(b)

```
4185148    777 STILALI-PKrtyak--emkdYRPISCcnvlYKAISKLlaNRLKCLLPEfia--pnQSAFISDRLl----------mENL 841
ApRT-like   47 DAIVVPIwKKkgskk--dcntYRGISLlshvGKMYAKIleRRTRAKTEHlls--daQFGFRKGRGc----------tDAI 112
903661     626 RGRTILI-PKkgdrg--dpsnYRPITClntcYKVLTSVmnSVILSHLSRgealpmnQRAMRKREWg----------cTHA 692
120551     397 LARTVFI-PKtvtak--rpqdFRPISVpsvlVRQLNAIlaTRLNSSINWdp----rQRGFLPTDGc----------aDNA 459
33301425   337 ASRTIMI-PKpgksdysdpssWRPITItsavYRLLMKYltWELYNWILLn------QMLSRSQKSlgkfegchdhnaMLN 409

                                          ######
4185148    842 LLASELvkdyhkd----glsprcaMKIDLSKAFDSVQWPFLLNTLaaldipekfihwin---------------lcista 902
ApRT-like  113 FALRQLceraley-----dkdlhlVFVDQEKAFDRVNRNKLWKILeqydikgqlldkiraiyan----srsavrttsgts 183
903661     693 MVLDRAmvmdamaq---kkhslsvAWLDYRKAYDSVSHEYIRWAInsvniprsvqltlkrlmsdwetrfestqcrpklrs 769
120551     460 TIVDLVlrhshkh-----frscyiANLDVSKAFDSLSHASIYDTLraygapkgfvdyvqntyeg----ggtslngdgwss 530
33301425   410 MLIQDVrrqtnpsnpinknkrlyiVFLDFTNAFGSVPLDTLMYVPqrfglgtsaltliknlyld----nytnvtcgeski 485

                         ##                                            # ##
4185148    903 sfsvqvnGLRQGCSLSPYLFVICMNVLsamldkgavekrf-gyhprcrnmgltHLCFADDIMVFsagsahslegVLAIFK 981
ApRT-like  184 dwfpvtsGVRQGCNLSPLLFVIYMDqitkean--------------pdpeslnELMFADDLAIInnkt--qlqeHINQLN 247
903661     770 dkmkvlnGIFQGDSLSPTLFVLCIAPIsyalnkgvgqcqsssgwsagygfeigHQFYMDDLKLYartpa-mldsQIQVVS 848
120551     531 eefvparGVKQGDPLSPILFNLVMDRLlrtlpseig--------akvgnaitnAAAFADDLVLFaetrm-glqvLLDKTL 601
33301425   486 envklnkGVKQGCPLSMLLFNIFINIIiraieampdvh-----gyplgdmdirILAYADDIALIs--------dSHKDLQ 552

                                                                            ##
4185148    982 DFaa-------fsGLNISLEKSTlfmasissetcas---------ilarfpfdsgslpVRYLGLPL 1031
ApRT-like  248 ASce-------kyDMKISISKTEvmtisrrpgkvdi----------ningsqlkqsreFKYLGSIF 296
903661     849 EVse-------amGLHLNLSKCAkahyaphgaggaqeavegaegsrkgeipilglrstYKYLGVEQ 907
120551     602 DFls-------ivGLKLNADKCFtvgikgqpkqkctvleaqsfyvgsseipslkrtdeWKYLGINF 660
33301425   553 EMvykaeyigrilGLLFNPSKCAlmdiphdkkrtppil------vngemikcvgkadpYKYLGTFR 612
```

Figure S2.

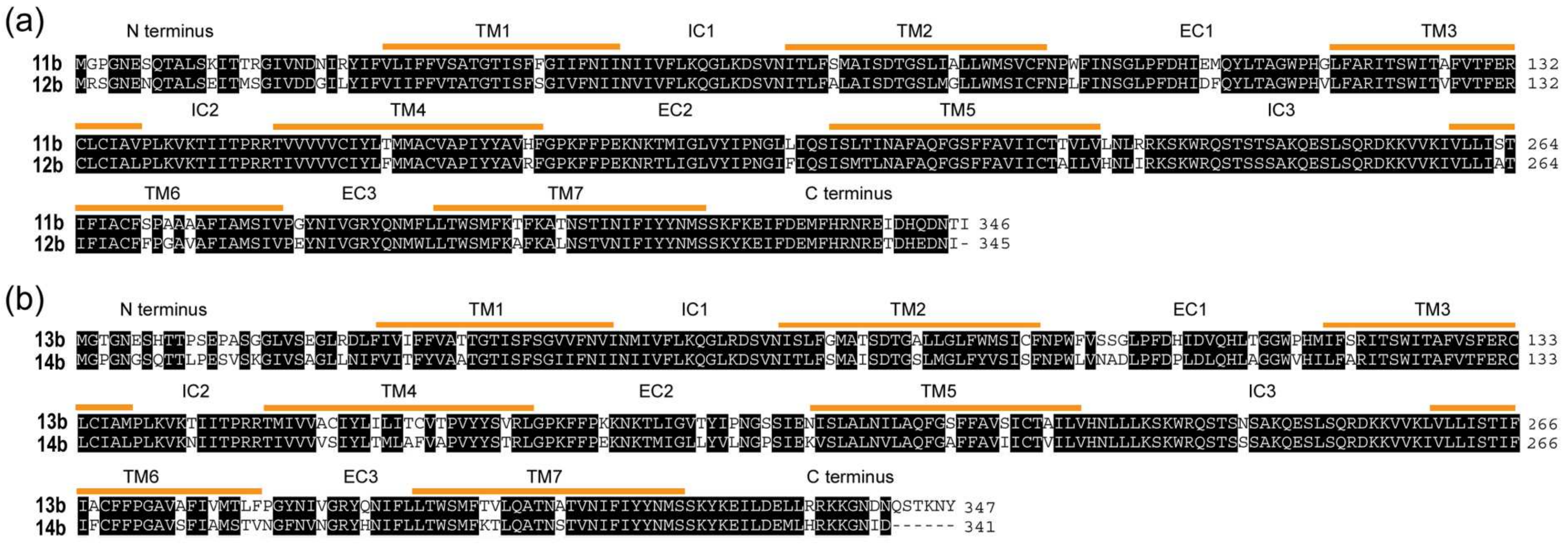
(a)
N terminus
TM1
IC1
TM2
EC1
TM3
11b MGPGNESQTALSKITTRGIVNDNIRYIFVLIFFVSATGTISFFGIIFNIINIIVFLKQGLKDSVNITLFSMAISDTGSLIALLWMSVCFNPWFINSGLPFDHIEMQYLTAGWPHGLFARITSWITAFVTFER 132
12b MRSGNENQTALSEITMSGIVDDGILYIFVIIFFVTATGTISFSGIVFNIINVIVFLKQGLKDSVNITLFALAISDTGSLMGLLWMSICFNPLFINSGLPFDHIDFQYLTAGWPHVLFARITSWITVFVTFER 132
IC2
TM4
EC2
TM5
IC3
11b CLCIAVPLKVKTIITPRRTVVVVVCIYLTMMACVAPIYYAVHFGPKFFPEKNKTMIGLVYIPNGLLIQSISLTINAFAQFGSFFAVIICTTVLVLNLRRKSKWRQSTSTSAKQESLSQRDKKVVKIVLLIST 264
12b CLCIALPLKVKTIITPRRTIVVVVCIYLFMMACVAPIYYAVRFGPKFFPEKNRTLIGLVYIPNGIFIQSISMTLNAFAQFGSFFAVIICTAILVHNLIRKSKWRQSTSSSAKQESLSQRDKKVVKIVLLIAT 264
TM6
EC3
TM7
C terminus
11b IFIACFSPAAAAFIAMSIVPGYNIVGRYQNMFLLTWSMFKTFKATNSTINIFIYYNMSSKFKEIFDEMFHRNREIDHQDNTI 346
12b IFIACFFPGAVAFIAMSIVPEYNIVGRYQNMWLLTWSMFKAFKALNSTVNIFIYYNMSSKYKEIFDEMFHRNRETDHEDNI- 345
(b)
N terminus
TM1
IC1
TM2
EC1
TM3
13b MGTGNESHTTPSEPASGGLVSEGLRDLFIVIFFVATTGTISFSGVVFNVINMIVFLKQGLRDSVNISLFGMATSDTGALLGLFWMSICFNPWFVSSGLPFDHIDVQHLTGGWPHMIFSRITSWITAFVSFERC 133
14b MGPGNGSQTTLPESVSKGIVSAGLLNIFVITFYVAATGTISFSGIIFNIINIIVFLKQGLKDSVNITLFSMAISDTGSLMGLFYVSISFNPWLVNADLPFDPLDLQHLAGGWVHILFARITSWITAFVTFERC 133
IC2
TM4
EC2
TM5
IC3
13b LCIAMPLKVKTIITPRRTMIVVACIYLILITCVTPVYYSVRLGPKFFPKKNKTLIGVTYIPNGSSIENISLALNILAQFGSFFAVSICTAILVHNLLLKSKWRQSTSNSAKQESLSQRDKKVVKLVLLISTIF 266
14b LCIALPLKVKNIITPRRTIVVVVSIYLTMLAFVAPVYYSTRLGPKFFPEKNKTMIGLLYVLNGPSIEKVSLALNVLAQFGAFFAVIICTVILVHNLLLKSKWRQSTSSSAKQESLSQRDKKVVKIVLLISTIF 266
TM6
EC3
TM7
C terminus
13b IACFFPGAVAFIVMTLFPGYNIVGRYQNIFLLTWSMFTVLQATNATVNIFIYYNMSSKYKEILDELLRRKKGNDNQSTKNY 347
14b IFCFFPGAVSFIAMSTVNGFNVNGRYHNIFLLTWSMFKTLQATNSTVNIFIYYNMSSKYKEILDEMLHRKKGNID------ 341

Figure S3.

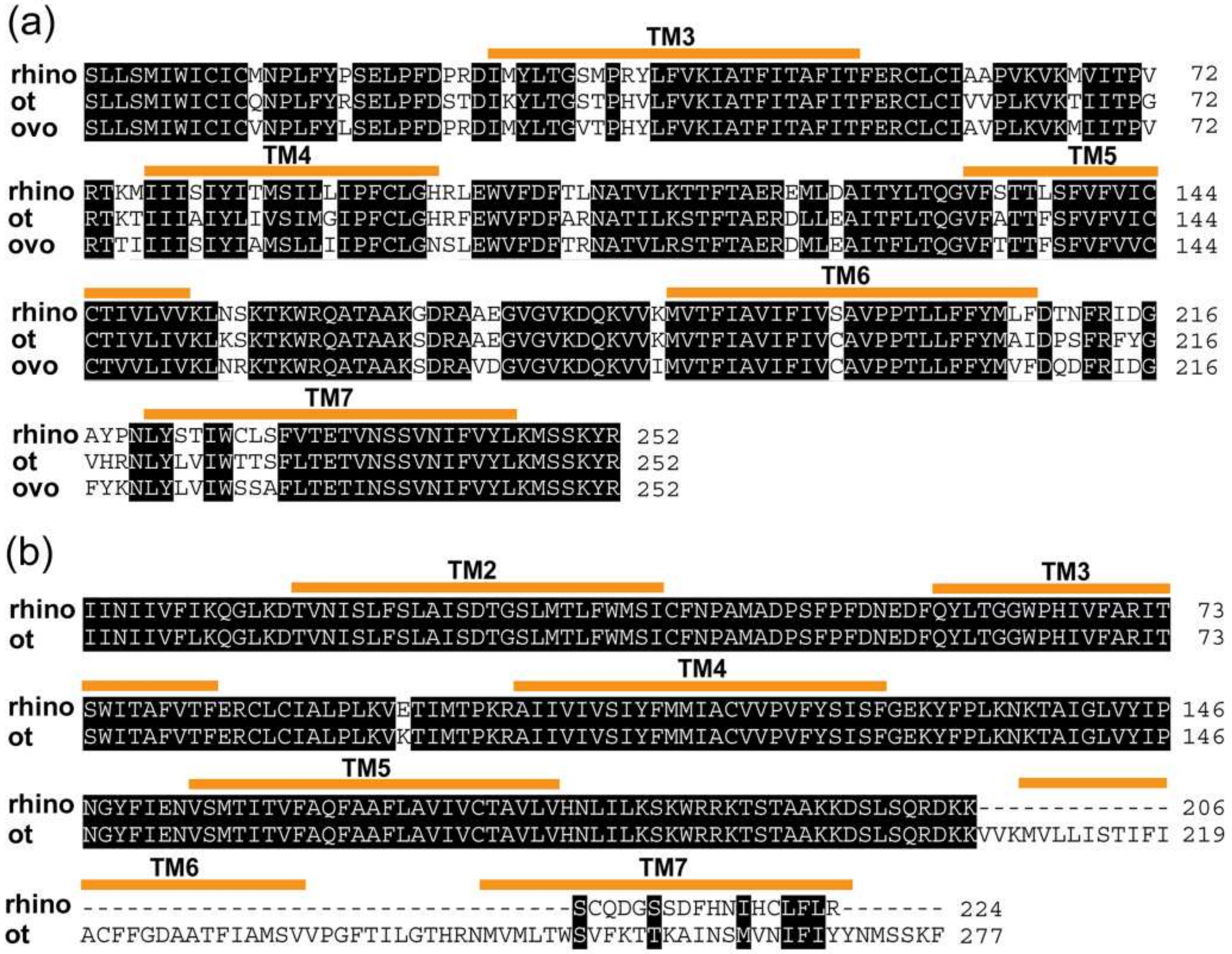
(a)
TM3
rhino SLLSMIWICICMNPLFYPSELPFDPRDIMYLTGSMPRYLFVKIATFITAFITFERCLCIAAPVKVKMVITPV 72
ot SLLSMIWICICQNPLFYRSELPFDSTDIKYLTGSTPHVLFVKIATFITAFITFERCLCIVVPLKVKTIITPG 72
ovo SLLSMIWICICVNPLFYLSELPFDPRDIMYLTGVTPHYLFVKIATFITAFITFERCLCIAVPLKVKMIITPV 72
TM4
TM5
rhino RTKMIIISIYITMSILLIPFCLGHRLEWVFDFTLNATVLKTTFTAEREMLDAITYLTQGVFSTTLSFVFVIC 144
ot RTKTIIIAIYLIVSIMGIPFCLGHRFEWVFDFARNATILKSTFTAERDLLEAITFLTQGVFATTFSFVFVIC 144
ovo RTTIIIISIYIAMSLLIIPFCLGNSLEWVFDFTRNATVLRSTFTAERDMLEAITFLTQGVFTTTFSFVFVVC 144
TM6
rhino CTIVLVVKLNSKTKWRQATAAKGDRAAEGVGVKDQKVVKMVTFIAVIFIVSAVPPTLLFFYMLFDTNFRIDG 216
ot CTIVLIVKLKSKTKWRQATAAKSDRAAEGVGVKDQKVVKMVTFIAVIFIVCAVPPTLLFFYMAIDPSFRFYG 216
ovo CTVVLIVKLNRKTKWRQATAAKSDRAVDGVGVKDQKVVIMVTFIAVIFIVCAVPPTLLFFYMVFDQDFRIDG 216
TM7
rhino AYPNLYSTIWCLSFVTETVNSSVNIFVYLKMSSKYR 252
ot VHRNLYLVIWTTSFLTETVNSSVNIFVYLKMSSKYR 252
ovo FYKNLYLVIWSSAFLTETINSSVNIFVYLKMSSKYR 252
(b)
TM2
TM3
rhino IINIIVFIKQGLKDTVNISLFSLAISDTGSLMTLFWMSICFNPAMADPSFPFDNEDFQYLTGGWPHIVFARIT 73
ot IINIIVFLKQGLKDTVNISLFSLAISDTGSLMTLFWMSICFNPAMADPSFPFDNEDFQYLTGGWPHIVFARIT 73
TM4
rhino SWITAFVTFERCLCIALPLKVETIMTPKRAIIVIVSIYFMMIACVVPVFYSISFGEKYFPLKNKTAIGLVYIP 146
ot SWITAFVTFERCLCIALPLKVKTIMTPKRAIIVIVSIYFMMIACVVPVFYSISFGEKYFPLKNKTAIGLVYIP 146
TM5
rhino NGYFIENVSMTITVFAQFAAFLAVIVCTAVLVHNLILKSKWRRKTSTAAKKDSLSQRDKK------------- 206
ot NGYFIENVSMTITVFAQFAAFLAVIVCTAVLVHNLILKSKWRRKTSTAAKKDSLSQRDKKVVKMVLLISTIFI 219
TM6
TM7
rhino ---------------------------------SCQDGSSDFHNIHCLFLR------- 224
ot ACFFGDAATFIAMSVVPGFTILGTHRNMVMLTWSVFKTTKAINSMVNIFIYYNMSSKF 277

Figure S4.

(a)

```
5'-GAGAGGGCGGGGTTTAAAAAAAAATAGAGAGTCTTTGTAAAGAGGTAAGCCAGTACAT
   GTAGATCTACCAGTATATTGTATTTGATAAGTCGTAAGCGCACGTTCAAGCCAAA
 113 atgccattatctaggttgtggactaatcaagaggtttattctcct
   1  M  P  L  S  R  L  W  T  N  Q  E  V  Y  S  P
 158 gatgggaacgagaccgcgttaatggcgacaaccacaggttctagt
  16  D  G  N  E  T  A  L  M  A  T  T  T  G  S  S
 203 ctttcgtcaacagacatacttagcgacgaggtgaccaactctgtg
  31  L  S  S  T  D  I  L  S  D  E  V  T  N  S  V
 248 gtcatctgtggtcttctcagtctactcggtgtgctgaccaacgtc
  46  V  I  C  G  L  L  S  L  L  G  V  L  T  N  V
 293 atcaacattgtggtgtttgccaagcaagggtttcaagacagcatg
  61  I  N  I  V  V  F  A  K  Q  G  F  Q  D  S  M
 338 aacattagtctcatgggactagcagtatctgacctgagctcactg
  76  N  I  S  L  M  G  L  A  V  S  D  L  S  S  L
 383 gtgaccatgatctggctcagtatattatgttacaagccgctttttc
  91  V  T  M  I  W  L  S  I  L  C  Y  K  P  L  F
 428 tacctctctgagttgccctttgaccccagggatatcatgtacctc
 106  Y  L  S  E  L  P  F  D  P  R  D  I  M  Y  L
 473 acggggtctacaacccattccgtctttgtcaaaatcgccacttta
 121  T  G  S  T  T  H  S  V  F  V  K  I  A  T  L
 518 atcaccgcctttatcaccttcgaacgatgtctgtgtatcgctgtc
 136  I  T  A  F  I  T  F  E  R  C  L  C  I  A  V
 563 cctctgaaggtgaagacgatcatcacacctggaaggacaaagaca
 151  P  L  K  V  K  T  I  I  T  P  G  R  T  K  T
 608 attattatctccatctaccttgccatatctcttttgatgatcccc
 166  I  I  I  S  I  Y  L  A  I  S  L  L  M  I  P
 653 ttcttccttggaaacagacttgaatgggttttgatttcagaacg
 181  F  F  L  G  N  R  L  E  W  V  F  D  F  R  T
 698 aatgccacagtgctaaaagcgacgtataaggccaagagagaaata
 196  N  A  T  V  L  K  A  T  Y  K  A  K  R  E  I
 743 ttagaagccattacgtttctcgcccaaggagcatttgcaacgacg
 211  L  E  A  I  T  F  L  A  Q  G  A  F  A  T  T
 788 ttttctttcgtcttcgtcatttgctgtacaatcgttcttgtcgtc
 226  F  S  F  V  F  V  I  C  C  T  I  V  L  V  V
 833 aaactcaacagtaaaacaaaatggcgaaaggctactgcagccaag
 241  K  L  N  S  K  T  K  W  R  K  A  T  A  A  K
 878 tctgatcgtgcaacggatggagttggggtcaaagatcaaaaggtt
 256  S  D  R  A  T  D  G  V  G  V  K  D  Q  K  V
 923 gtgaaaatggtaaccttcattgctgtcatattcatcgtctgctcc
 271  V  K  M  V  T  F  I  A  V  I  F  I  V  C  S
 968 gtgccacccacgctcgtgtttttgtatatggtgtttgacacagat
 286  V  P  P  T  L  V  F  L  Y  M  V  F  D  T  D
1013 tttcgtattgacggtgtctatcgaaatctcttccttgtcgtttgg
 301  F  R  I  D  G  V  Y  R  N  L  F  L  V  V  W
1058 tctacaacatttcttacagaaacgataaactccagcgtgaacata
 316  S  T  T  F  L  T  E  T  I  N  S  S  V  N  I
1103 tttgtgtatctgaaaatgagttcgaaataccgagctgtgtttatg
 331  F  V  Y  L  K  M  S  S  K  Y  R  A  V  F  M
1148 aaaacattttttgaacaagcaggaaaga
 346  K  T  F  L  N  K  Q  E  R
TAACTTTTAAACTAAAAAAGAAAATGTATTCTGTTAGATAATAAACGAAAACATAAT
AATACTTGCGAAAAAAAAAAAAAAAAAAAAAAAAAAAAA-3'
```

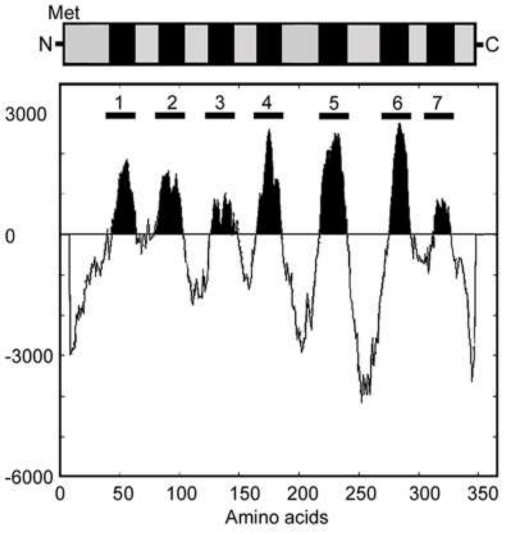

(b)

```
5'-CTTCTGTTGAACCGATAATCCTGTATAATACGGAATGACAGGGAGGAGGAAAAAGTA
   TACAAATATTGACTTCCTATATCAGTTTAACACGAGCGAATAAAACAATCATTGACA
   ATTTGACTTATTTTGGAAACAACGTTGTGTTACGATTTTTCCAGCAAACAACATAGT
   TATGAAATAGGTTTAATGCCCTCTTATAAAAGTCTGCACTGAATGACAAGACCCCTG
   GTGGACTATTATCTCTGTAAAAAAATCATTAAACCGTTTTGAAAGAAACAGAAAATG
   CGCACGTAACAGTGCAAATCTACAGCTTTGAAAAGCGCTAATCTAGCGGAACCCGAA
 343 atgggccccgggaacaacagtcaggcaagatcctcaaagagcaca
   1  M  G  P  G  N  N  S  Q  A  R  S  S  K  S  T
 388 cagaaaggtttgttggacgactacacactcgctgtcctctcactg
  16  Q  K  G  L  L  D  D  Y  T  L  A  V  L  S  L
 433 attctgtacgtgtttgctatcgggatcgtcagcgtttacggcctc
  31  I  L  Y  V  F  A  I  G  I  V  S  V  Y  G  L
 478 atctgcaacgtcatcaacatcatagttttctgtaagcagggcttc
  46  I  C  N  V  I  N  I  I  V  F  C  K  Q  G  F
 523 aaagataccgtaaacatcacattgttcgggctgaccatttcggat
  61  K  D  T  V  N  I  T  L  F  G  L  T  I  S  D
 568 atgggatgtgccatcactctgtttgggggagcgtgtgttttaac
  76  M  G  C  A  I  T  L  F  W  G  S  V  C  F  N
 613 cctctgtttatcgaagccgacctgcccatggtgtaccaagacata
  91  P  L  F  I  E  A  D  L  P  M  V  Y  Q  D  I
 658 atctatctcacgtcaggatggcctcttgtgtgttttgcgcgtatc
 106  I  Y  L  T  S  G  W  P  L  V  C  F  A  R  I
 703 tccagttggatcacagcttttgttacattcgagaggtgtctctgc
 121  S  S  W  I  T  A  F  V  T  F  E  R  C  L  C
 748 attactgtgcccctgaaggtgaagatgattctgacgccgaggaga
 136  I  T  V  P  L  K  V  K  M  I  L  T  P  R  R
 793 acagtcttcgtagttgtgggaatttacttgggcataattctttgc
 151  T  V  F  V  V  V  G  I  Y  L  G  I  I  L  C
 838 gtagtgccactgtattatgcaatgggactgggtccaagacatttt
 166  V  V  P  L  Y  Y  A  M  G  L  G  P  R  H  F
 883 ccggagagaaatgtaacgataattggatcggtttacaatgaaaac
 181  P  E  R  N  V  T  I  I  G  S  V  Y  N  E  N
 928 ggtcctttctatgagggagtagctcttacgcttagcgcgttttcc
 196  G  P  F  Y  E  G  V  A  L  T  L  S  A  F  S
 973 caactggcttcgtttttttgccgttatcatctgtactgggattctg
 211  Q  L  A  S  F  F  A  V  I  I  C  T  G  I  L
1018 gtccacaattttctcttgaaatcaaaatggcggcagtcggcttca
 226  V  H  N  F  L  L  K  S  K  W  R  Q  S  A  S
1063 agcgccacaagacaggaattttttaacaaacagagacaagaaagtt
 241  S  A  T  R  Q  E  F  L  T  N  R  D  K  K  V
1108 gtcaagatgattctcttcatttcaagtttattcatcgtgtttttc
 256  V  K  M  I  L  F  I  S  S  L  F  I  V  F  F
1153 tcaccgacggctgctaatacgtttgtcatgatgatcagttcggag
 271  S  P  T  A  A  N  T  F  V  M  M  I  S  S  E
1198 taccgcacgggaggcagataccaaaatgtttatctcctcaactgg
 286  Y  R  T  G  G  R  Y  Q  N  V  Y  L  L  N  W
1243 gccatttcttgtttgcttgtggggacaaactcgacggtgaatatc
 301  A  I  S  C  L  L  V  G  T  N  S  T  V  N  I
1288 tttgtctactacagcatgagctcaaagtacaggaaaattcttgac
 316  F  V  Y  Y  S  M  S  S  K  Y  R  K  I  L  D
1333 gaaatgctcaaaagaaaggaagggaacagagggccttcggagagg
 331  E  M  L  K  R  K  E  G  N  R  G  P  S  E  R
1378 aaggctgtacgctgtatccaatctttg
 346  K  A  V  R  C  I  Q  S  L
TGAACGTTATATATGCGTTGTCACAATATTATGATAGCTCAAATACAGCTTTCTGTT
CTATAGAAAAAAAAAAAAAAAA-3'
```

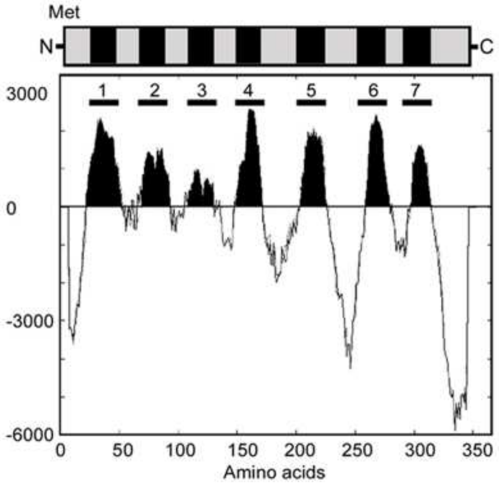

(c)

```
5'-AAATAAAATCGAGAAAGGGAAAGGGAGAGGAAAGGATAGGGGGTGAGGAAGAGGAAG
   AGGAAGAAGAAGAAGAAGAAGAAGAAGAAGAAGAAGAAGAAGAAGAAGAAGAAGAAG
   AAGAAGAAGAAGAAGAAGAAGAAGAAGAAGGAGAAGAAGAAGTTGGACCTATCCGCG
   GCACCCTCTAACCACCCACAACGTGTGCGCGCATGTTGGCTTCAGATATCGCGTTAT
   CAGTGCTGCATCTTACTGCGAGATTTAAGACTCATAGTGATCGATGTTCTTGAAGTC
   GTTACAAACTGGAAACCGTATTTGGCGTGTTTTTCGGGTACTTGTATGATTTTCATT
   ATGTGGTTTCAATCTCTTAATGTGTCTTGAGCTGTTGTTGTTTTTTTACTCAGAAAG
   TCTCCCGGCTAATCTGGTGTCACAATTATTTCTCCGTAGCCTATCCACTATCCACCA
   ATTTCAAAAGACACGATT
     475 atgtcccttgtcaacgagacagaacgggagctcaagggatcccac
     1    M  S  L  V  N  E  T  E  R  E  L  K  G  S  H
     520 aacatctctgaacatggcggcttaattgacgaccaaactttgcgt
     16   N  I  S  E  H  G  G  L  I  D  D  Q  T  L  R
     565 attttcctacttgtgttcacctttgtcaccctaggtctcagcctt
     31   I  F  L  L  V  F  T  F  V  T  L  G  L  S  L
     610 ttaggctcggcgttcaactgtataaacatcactgtgttcttgacg
     46   L  G  S  A  F  N  C  I  N  I  T  V  F  L  T
     655 ctgggagctaaggactgtgtgtctgtctgtctcctttcacttgcc
     61   L  G  A  K  D  C  V  S  V  C  L  L  S  L  A
     700 gtttccgatttcacatgcctgtttctcggggctgtttgcggagtt
     76   V  S  D  F  T  C  L  F  L  G  A  V  C  G  V
     745 tgtgacatactcgatgcctatggctctgctgacttctatgtggat
     91   C  D  I  L  D  A  Y  G  S  A  D  F  Y  V  D
     790 ccccgagggctatattaccaagtaatattcatcagttcaatgtca
     106  P  R  G  L  Y  Y  Q  V  I  F  I  S  S  M  S
     835 tatgacatttcgacctatatcacagccttcatctcactggaacgt
     121  Y  D  I  S  T  Y  I  T  A  F  I  S  L  E  R
     880 tgcctctgcgtggctttacctttccgtttcaaagaactgttcacc
     136  C  L  C  V  A  L  P  F  R  F  K  E  L  F  T
     925 tttaaacgagccgtattggctatggcgacaatcttttgcctcaca
     151  F  K  R  A  V  L  A  M  A  T  I  F  C  L  T
     970 ttctgctgttacctccccattacgtcacatccggtctccgtgtc
     166  F  C  C  Y  L  P  H  Y  V  T  S  G  L  R  V
    1015 cagtgggaccccggacgaacacgacccgcgtcctcctgtggagc
     181  Q  W  D  P  R  T  N  T  T  R  V  L  L  W  S
    1060 tcgaaagacatgccggccatcacagctttcttagatttgtggaac
     196  S  K  D  M  P  A  I  T  A  F  L  D  L  W  N
    1105 catctgattctagcggtcacttcagtggtcatcgtcattgtgtgt
     211  H  L  I  L  A  V  T  S  V  V  I  V  I  V  C
    1150 acccacatcatggtcaccggcctcaagaaatcatctcagttccag
     226  T  H  I  M  V  T  G  L  K  K  S  S  Q  F  Q
    1195 agacgcggagctgcgagaccgtcagagccagatggtccgaacaat
     241  R  R  G  A  A  R  P  S  E  P  D  G  P  N  N
    1240 ttcaaaacttcgaacgaagtaggagaaggagaagaaaacgttttg
     256  F  K  T  S  N  E  V  G  E  G  E  E  N  V  L
    1285 agagacccagacagtcgcagagataacaatatttccacgccgtat
     271  R  D  P  D  S  R  R  D  N  N  I  S  T  P  Y
    1330 tgtcccacaaatgtggaaaaagaaagtagtaacaagattaaagtc
     286  C  P  T  N  V  E  K  E  S  S  N  K  I  K  V
    1375 gagaaaagtccccaaactctgtcggccaaaaatcgacgtgttgtg
     301  E  K  S  P  Q  T  L  S  A  K  N  R  R  V  V
    1420 aaaatggtatccacactggcaatcgtctccatcttgtgcaatacg
     316  K  M  V  S  T  L  A  I  V  S  I  L  C  N  T
    1465 tcacgacttctgttcgtggtggccctgagagctgagcccgacata
     331  S  R  L  L  F  V  V  A  L  R  A  E  P  D  I
    1510 aacttcggacatcgttatcacaacttgtacatggtcatcctggtc
     346  N  F  G  H  R  Y  H  N  L  Y  M  V  I  L  V
    1555 ttggcctatatctttcaagttatcaatgctcctgtcaacattttt
     361  L  A  Y  I  F  Q  V  I  N  A  P  V  N  I  F
    1600 atatacctcaagctaaatccgtcgtacagaaaaacattctctcag
     376  I  Y  L  K  L  N  P  S  Y  R  K  T  F  S  Q
    1645 atttttggcattggtcagacaaaa
     391  I  F  G  I  G  Q  T  K
   TAAAATCAGACTTTTTAGAAAGTTCTCCTATTCGCTCAATATTGTAAGAATGGCGTA
   AAAAAAAAAAAAAAAAAAAAAAAAAAA-3'
```

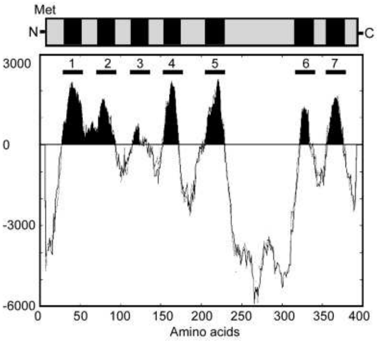

Target subfamily		Sense primer 5′ → 3′	Antisense primer 5′ → 3′
a	A1	TCTGCGTGAACCCGCTGTTYTACCT	TCGRTATTTYGAACTCATTTTCAG
	A2	ATGGATAAGYATMTGCVWGAACCCG	TCGRTATTTYGAACTCATTTTCAG
	A3	TCCYTRCTGWCCATGATATGGATAT	TCGRTATTTYGAACTCATTTTCAG
	A4	TCTGCTGGAACCCACTCTTTTACAA	TCGRTATTTYGAACTCATTTTCAG
	A5	TCTGCSAGAACCCGCTTTTCTATCG	TCGRTATTTYGAACTCATTTTCAG
	A3′	CTTACAGAAACGATAAACTCCAGCG	ATTAACCCTCACTAAAGGGA (T3)
	A5′	GACCCCGTGAGGTACATGATATCC	TAATACGACTCACTATAGGG (T7)
b	B1	YCATCAGCTTYTTTGGCATTGTGGC	RWAYTTBGARCTCATNTWRTAATA
	B2	AACGYMRTCAACATSATMGTTTTC	RWAYTTBGARCTCATNTWRTAATA
	B3	CCGGYACCATCAGTTTCTTCGGTC	RWAYTTBGARCTCATNTWRTAATA
	B4	CATCATMAACATAATAGTGTTCWTA	RWAYTTBGARCTCATNTWRTAATA
	B5	MWYMRTCTGYTTMTTTGGCATTATA	RWAYTTBGARCTCATNTWRTAATA
	B6	GCARGGHTTAAAGGACAGTGTAAAT	RWAYTTBGARCTCATNTWRTAATA
	B7	GGYRYYATCTGCAACATCATCAACG	RWAYTTBGARCTCATNTWRTAATA
	B8	CRAAYATYMTCAACATCMTYGTG	RWAYTTBGARCTCATNTWRTAATA
	B3′	TTCTCACCGACGGCTGCTAATACG	ATTAACCCTCACTAAAGGGA (T3)
	B5′	AAATGGTCAGCCCGAACAATGTG	TAATACGACTCACTATAGGG (T7)
c	C1	GCTCGRYRTTYAACTGYATAAACAT	AGTRTGGAYACCATTTTCACAAC
	C2	TGYGTGTCYGYCTGCTTGCTGGC	GTTRGCMGAGGMGTTMAYKGTCTG
	C3	CRCMCTAGAACGMTGYCTBTGTGT	BAGGGCVGAYACNRTTTTGATCA
	C3′	TATTTCCACGCCGTATTGTCCCAC	AAGCAGTGGTATCAACGCAGAGT
	C5′	GCTCTAATACGACTCACTATAGG	CATAGAAGTCAGCAGAGCCATAGG
